# Noninvasive morpho-molecular imaging reveals early therapy-induced senescence in human cancer cells

**DOI:** 10.1126/sciadv.adg6231

**Published:** 2023-09-13

**Authors:** Arianna Bresci, Jeong Hee Kim, Silvia Ghislanzoni, Francesco Manetti, Lintong Wu, Federico Vernuccio, Chiara Ceconello, Salvatore Sorrentino, Ishan Barman, Italia Bongarzone, Giulio Cerullo, Renzo Vanna, Dario Polli

**Affiliations:** ^1^ Department of Physics, Politecnico di Milano, Milan, Italy.; ^2^ Department of Mechanical Engineering, Johns Hopkins University, Baltimore, MD, USA.; ^3^ Department of Advanced Diagnostics, Fondazione IRCCS Istituto Nazionale Tumori, Milan, Italy.; ^4^ Department of Oncology, Johns Hopkins University School of Medicine, Baltimore, MD, USA.; ^5^ The Russell H. Morgan Department of Radiology and Radiological Science, Johns Hopkins University School of Medicine, Baltimore, MD, USA.; ^6^ CNR-Institute for Photonics and Nanotechnologies (CNR-IFN), Milan, Italy.

## Abstract

Anticancer therapy screening in vitro identifies additional treatments and improves clinical outcomes. Systematically, although most tested cells respond to cues with apoptosis, an appreciable portion enters a senescent state, a critical condition potentially driving tumor resistance and relapse. Conventional screening protocols would strongly benefit from prompt identification and monitoring of therapy-induced senescent (TIS) cells in their native form. We combined complementary all-optical, label-free, and quantitative microscopy techniques, based on coherent Raman scattering, multiphoton absorption, and interferometry, to explore the early onset and progression of this phenotype, which has been understudied in unperturbed conditions. We identified TIS manifestations as early as 24 hours following treatment, consisting of substantial mitochondrial rearrangement and increase of volume and dry mass, followed by accumulation of lipid vesicles starting at 72 hours. This work holds the potential to affect anticancer treatment research, by offering a label-free, rapid, and accurate method to identify initial TIS in tumor cells.

## Teaser

Multimodal label-free optical microscopy detects therapy-induced senescence in tumor cells at an unprecedentedly early stage.

## INTRODUCTION

Cancer is among the leading threats to life worldwide, accounting for nearly 10 million deaths in 2020 (*1*). Standard clinical anticancer protocols exploit drugs (i.e., chemotherapy), ionizing radiation (i.e., radiotherapy), or a combination of these two to suppress tumor progression. Although they are based on different principles of interaction, both chemotherapy and radiotherapy aim to induce the death of cancer cells, mostly through apoptosis, by causing irreversible damage. However, over the past two decades, it has been observed that a small percentage of treated cancer cells becomes resistant to apoptosis, despite losing their proliferative capacity. They are known as therapy-induced senescent (TIS) cells and have been recognized as a central component of the response of cancer cells to therapy (*2*). At the cellular level, senescence is characterized by a progression of complex changes, including chromatin alterations and metabolic modifications (*3*). In the context of cancer treatment, senescence has been regarded for years as a co-adjuvant tumor-suppressive mechanism. Nevertheless, recent studies have shown that TIS cancer cells can eventually create a survival niche via paracrine cooperation with neighboring cells, potentially contributing to tumor dormancy, resistance to therapy, and disease recurrence (*4*). Not only do senescent cells secrete a variety of proinflammatory factors that can negatively affect neighboring, noncancerous cells; the senescent state can also be evaded, with cells returning to cell cycle and demonstrating a more aggressive phenotype (*5*). Reports show that such standardized anticancer treatments produce effective clinical outcomes in only 7% of cancer patients (*6*). To increase efficacy, further therapy screening is necessary to select optimized treatments that limit the insurgence of protumorigenic TIS phenotypes. Functional screening is acknowledged to produce more effective tumor therapy protocols than those developed through genomic data and to improve clinical outcomes (*7*). Ultimately, the growing field of precision functional oncology would exploit in vitro therapy screening on expanded tumor biopsy–derived cells. To these aims, several markers are commonly used to identify senescence activation in vitro (*8*, *9*). Morphological changes, such as enlarged and flattened cells, are common features of senescent cells, but show the least specificity. Markers, including SA-β-gal, p16, p21, H3K9me3, γH2AX, immunochemical and colorimetric assays, gene expression, and mass spectrometry (*10*), are widely used for the identification of senescence. However, such markers show varying degrees of false positivity and do not provide a comprehensive profile of the targeted senescence-associated phenotype, requiring a combination of various measurements for accurate identification. Furthermore, they would impair the fast and effective detection of TIS cells, due to their destructive and/or time-consuming nature.

Hence, it is crucial to develop simple and reproducible methods to noninvasively, rapidly, and accurately distinguish and comprehensively describe the onset and progression of TIS in human cancer cell cultures in their native state, based on both the biochemical and the morphological standpoints (*11*). A possible solution to this problem comes from optical techniques, such as label-free vibrational spectroscopy and microscopy (*12*), capable of measuring the vibrational spectrum of the molecules composing the specimen with subcellular spatial resolution. This provides an endogenous signature that can be used as a fingerprint for the unique characterization of their chemical content in terms of lipids, proteins, nucleic acids, carbohydrate concentration, and many more. The vibrational spectrum can be accessed either directly, by measuring the absorption spectrum of the specimen in the infrared spectral region, e.g., via Fourier transform infrared spectroscopy, or indirectly, via the (spontaneous) Raman (SR) effect, which provides higher spatial resolution. The Popp group used these two techniques to study senescence induced by long-term cell cultivation in single human fibroblast cells (*13*). They identified senescence-associated biomolecular changes: Nucleic acids and proteins were slightly down-regulated, while lipids were up-regulated in aged fibroblasts. Similarly, Bai *et al.* (*14*) acquired SR signatures of mesenchymal stem cells obtained from the human umbilical cord during replicative senescence by serial passaging. The authors found that the ratio of Raman peaks associated with protein vibrations could serve as a marker for the senescent phenotype. However, these works focused on replicative senescence, not TIS. In this aspect, TIS induced by doxycycline treatment was studied in label-free MCF-7/NeuT human breast cancer cells by Mariani *et al.* (*15*) using SR microscopy. This study reported that the spectra of nuclei from senescent cells showed different peaks, suggesting instabilities in the nuclear membrane as indicative of TIS. In addition to vibrational spectroscopy, linear (i.e., single-photon) endogenous fluorescence is another label-free optical technique that can be used to investigate cell states. It was used in combination with a flow cytometer by the Jones group, reporting that replicative senescent cells yielded a higher fluorescence signal with respect to normal human mesenchymal stem cells (*16*).

The aforementioned label-free linear optical techniques for chemical imaging feature relevant limitations and disadvantages: They suffer from low throughput and extended measurement time, thus discouraging a broad applicability in cancer therapy research, and/or cannot provide a full quantitative description of both the biochemical and morphological traits of TIS onset and its progression, especially if used individually. This calls for nonlinear optical (NLO) techniques (*17*, *18*) and multimodal imaging approaches (*17*). Using ultrashort laser pulses, NLO can extract more information in a shorter time because of the temporal confinement of the excitation, which entails higher laser intensity at the focus. Mainly working in the near-infrared spectral region, it also avoids direct (one-photon) absorption, thus preventing potential cell damage. The first investigation of TIS using NLO techniques was carried out by Oh *et al*. (*19*) by applying quantitative stimulated Raman scattering (SRS) (*20*) in an A7 malignant melanoma cell line. However, their results on human cancer cells were not consistent with the major senescence traits that were previously characterized via standard, destructive approaches and did not provide a full chronological description of initial TIS manifestation. Although previous studies have demonstrated the potential of NLO techniques as a promising tool to study senescence, specifically TIS cells, their scope was limited and did not cover comprehensive stages of TIS. Thus, further analysis must be conducted to exploit the advantages of these techniques. Furthermore, despite efforts in understanding senescence, no single marker specific to TIS in cancer environment has yet been identified to facilitate the rapid and accurate detection of TIS cells at an early stage.

To address this need, multimodal imaging methods can be used to elucidate this complex and enigmatic cell state. Safe and noninvasive methods without requiring any extended cell manipulation will enable us to identify general cellular traits, which can be directly correlated with TIS progression. Discovering such traits of TIS cells is critical in advancing current understanding of controversial TIS cells, thus enhancing widely accepted and established protocols for anticancer therapy by closely monitoring the risk of tumor relapse and resistance to therapy. In addition to chemical mapping through NLO techniques, interferometry-based quantitative phase imaging (QPI) (*21*, *22*) can offer complementary morphological information to chemical imaging. QPI emerged as a label-free linear optical method that rapidly generates three-dimensional (3D) maps of the cellular refractive index (RI). QPI allows the quantification of a number of cellular and subcellular characteristics, such as dry mass, volume, surface, and thickness, in an entirely noninvasive manner, by exploiting the fact that the optical phase shift of a laser beam through a specimen contains information about its RI variations due to structural features (*23*). In the context of cell senescence research, it has been exploited to distinguish subsets of human senescent T cells through a cytometry platform for tomographic imaging (*24*).

**Fig. 1.**
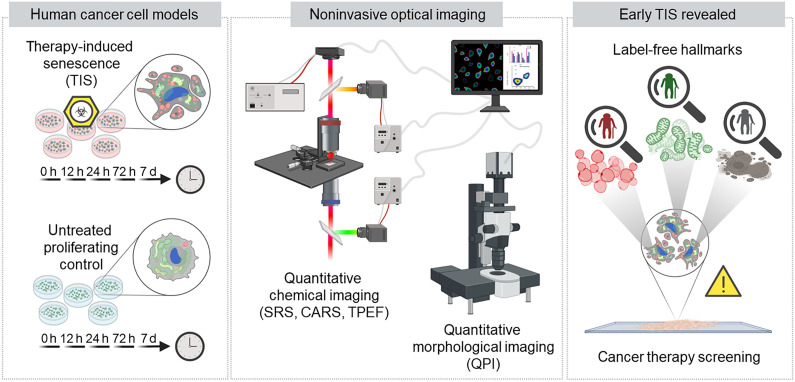
Schematic illustration of the research design. Human cancer cells from the HepG2 line were used as cellular models to investigate morpho-molecular traits of TIS onset and manifestation. In this part of the study, senescence was induced through treatment with 100 μM deferoxamine (DFO) (*25*). Five different TIS cultures and five control counterparts were prepared for each different time point and monitored over the treatment time. Noninvasive all-optical imaging tools [coherent anti-Stokes Raman scattering (CARS), stimulated Raman scattering (SRS), and two-photon excited fluorescence (TPEF)] were used to record biochemical quantitative data of unperturbed samples. Quantitative phase imaging (QPI) recorded quantitative morphological data. Statistically significant traits emerging from label-free measurements constitute a powerful tool for early detection (~72 hours via lipid alterations probed via SRS, ~24 hours via mitochondrial rearrangements probed with TPEF, ~24 hours via cell enlargement and flattening, and dry mass increase, probed via QPI) of TIS cells in anticancer therapy screening.

Here, we demonstrate that multimodal NLO microscopy and QPI can characterize both the chemical and morphological traits of TIS across early-to-late stages in human cancer cells without the use of any exogenous labels, as described in Fig. 1. Our study is conducted based on a validated cellular model of senescence proposed by Ghislanzoni *et al.* (*25*), which exploits deferoxamine (DFO)–mediated iron dysregulation with consequent mitochondrial dysfunction, as a trigger for the development of the TIS phenotype (see note S1). By analyzing a large population of such TIS cells, we could overcome the intrinsic, high biological variability of these experiments. Hence, we propose a combination of quantitative hallmarks of early- and late-stage TIS, detectable noninvasively via NLO and QPI techniques, targeting mitochondrial damage, lipid droplet accumulation (*26*, *27*), and morphological reshaping (*17*, *28*). In particular, we use a combination of co-registered NLO approaches for chemical characterization: two-photon excited fluorescence (TPEF) and coherent Raman scattering, in both the SRS and coherent anti-Stokes Raman scattering (CARS) modalities (*29*), exploiting the higher imaging speed and spatial resolution that stimulated Raman allows with respect to its spontaneous counterpart (*30*). Also, we quantitatively probe senescent features with respect to cell morphology and physiology through QPI, visualizing phase delays arising from the intrinsic cell structure. Coupling NLO microscopy with QPI enabled high-speed and high-resolution TIS imaging, in its unperturbed manifestation, at an unprecedented stage of phenotype commitment. The combined imaging techniques that are used to identify TIS hallmarks in this study offer a rapid, safe, and accurate detection of TIS within human tumoral cultures (*31*, *32*), fostering advancements in anticancer therapy research and in vitro TIS screening, as well as in further preclinical testing using patient-derived cancer cell cultures.

## RESULTS

### Multimodal NLO imaging unveils subcellular chemical maps of TIS cells

**Fig. 2.**
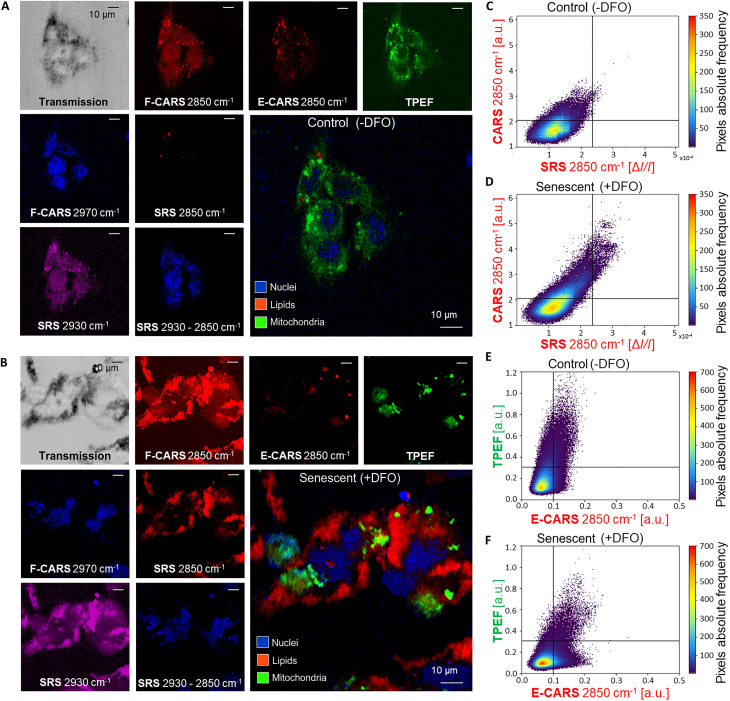
Multimodal NLO imaging reveals subcellular components in human cancer cells. (**A** and **B**) Representative images of control and senescent HepG2 induced by a 72-hour DFO treatment, showing all relevant NLO channels for the identification of subcellular mass. (**C** and **D**) Colocalization graphs of CARS and SRS signals. Crosshairs threshold lipid droplets from low-density lipid contributions and background (see Materials and Methods). CARS colocalization with SRS (M1) increases in TIS cells compared to controls (*P* = 0.0049), suggesting lipid droplet accumulation. (**E** and **F**) Colocalization graphs of E-CARS and TPEF. Crosshairs threshold the mitochondrial network, in the TPEF channel, and subwavelength CH-stretching concentrations, in the E-CARS channel, from the background (see Materials and Methods). M1 decreases in TIS cells (*P* = 5.8 × 10^−3^), indicating the accumulation of small cytoplasmic lipid vesicles unrelated to mitochondria. M2 increases in TIS cells (*P* = 1.9 × 10^−3^), suggesting the shrinking of mitochondrial constituents in dense spots, including mitochondrial lipids.

We observed both untreated proliferating HepG2 human hepatic cancer cells (Fig. 2A) and TIS senescent counterparts (Fig. 2B), in paraformaldehyde (PFA)–fixed conditions, via multimodal NLO microscopy. This cell line acts as a model system for the study of TIS through the use of DFO as a senescence-inducing agent. High-resolution multichannel images include co-registered SRS, TPEF, forward-detected CARS (F-CARS), epi-detected CARS (E-CARS), and linear light transmission modalities.

When matching the 2850 cm^−1^ Raman mode of CH_2_ stretching in lipids, F-CARS images show a strong signal in correspondence to lipid vesicles, which are dense fat storage organelles, consisting of neutral lipids and cholesterol esters, with phospholipids and diffused cytosolic lipids providing a minor contribution (refer to note S3 for reference SR spectra measured in cellular lipid standards). Because of spurious four-wave mixing processes unrelated to resonant molecular vibrations, the F-CARS signal of lipid droplets was embedded in a nonresonant background (NRB) produced by the whole organic matter. Nevertheless, the higher concentration of CH_2_ bonds in vesicles allowed us to distinguish them as brighter F-CARS spots, with the NRB providing an intrinsic self-heterodyne amplification of their vibrational signal. The co-registered NRB-free SRS channel was fundamental to confirm the location of these lipid accumulations, though with a lower contrast due to the modulation-transfer detection that implies a poorer signal-to-noise ratio with respect to CARS (see the “Multimodal NLO microscopy” section in Materials and Methods). Observing the co-occurrence of SRS and F-CARS signals at 2850 cm^−1^ frequency, one can quantitatively check the vibrational origin of F-CARS and corroborate the information about lipid vesicles (Fig. 2, C and D) (see the “NLO channels colocalization analysis” section in Materials and Methods). Both control cells (Fig. 2C) and TIS cells (Fig. 2D) showed a fairly strong positive correlation between 2850 cm^−1^ SRS and F-CARS, according to the Pearson correlation coefficient (PCC), reported in Table 1. Approximately 100% of SRS signals were colocalized with high F-CARS intensity values in both untreated and TIS cells, according to Manders’ colocalization coefficient (*33*), M1 in Table 1. Conversely, colocalization dropped when considering the fraction of F-CARS signal overlapping with SRS [refer to M2 (*33*) values in Table 1]. The colocalization analysis implies that most contributions to CARS in control cells originate from spurious NRB and NRB-amplified signal from small lipid accumulations, which in turn do not generate evident SRS. Overall, a disproportionate lipid accumulation is evident in senescent cells in the form of an elongated, uprising data cloud (Fig. 2D), which is evidently absent in controls (Fig. 2C): Dense lipid vesicles produce intense SRS signal colocalized with F-CARS in TIS phenotypes. Abundant lipid accumulations are qualitatively evident in both SRS and F-CARS channels of the NLO images of TIS cells (Fig. 2B), clearly indicating lipid overexpression compared to proliferating cancer cells (Fig. 2A).

**Table 1.** Quantitative colocalization metrics for multimodal NLO image channels. Pearson correlation coefficient (PCC) and Manders’ colocalization coefficients (M1 and M2) for the quantification of co-occurrence of NLO signals (2850 cm^−1^ CARS versus 2850 cm^−1^ SRS from lipid aggregates, and 2850 cm^−1^ CARS and TPEF for mitochondrial compounds) on a pixel-wise scale (<350 nm). Values displayed as mean ± SD.

	2850 cm^−1^ CARS and 2850 cm^−1^ SRS		2850 cm^−1^ E-CARS and TPEF	
	Control	TIS	Control	TIS
PCC	0.706 ± 0.068	0.672 ± 0.021	0.688 ± 0.025	0.652 ± 0.028
M1	0.996 ± 0.003	0.993 ± 0.003	0.678 ± 0.045	0.441 ± 0.056
M2	0.027 ± 0.009	0.167 ± 0.036	0.630 ± 0.039	0.830 ± 0.034

Endogenous TPEF highlighted dense gatherings of nicotinamide adenine dinucleotide [NAD(P)H] and flavin adenine dinucleotide (FAD), major mitochondrial coenzymes (Fig. 2, A and B). To corroborate the mitochondrial origin of such signal, we measured E-CARS signals at 2850 cm^−1^. The peculiar counter-propagating direction of the E-CARS radiation with respect to the excitation fields allowed us to suppress the NRB and distinguish scatterers with subwavelength axial length (*34*). E-CARS features a large wave-vector mismatch, which serves as a size filter that rejects the signal from thick elements and aqueous medium, allowing high-sensitivity imaging of small objects (*35*). At the 2850 cm^−1^ Raman mode, E-CARS channels displayed small-thickness components featuring substantial CH_2_-stretching modes. Consistently, we observed that these subwavelength subcellular compounds could not be detected via either F-CARS or SRS, as signals were overwhelmed by NRB or submerged by noise, respectively, but they were visible in the E-CARS modality. Colocalization analyses between TPEF and 2850 cm^−1^ E-CARS denote strong pixel-wise (<350 nm) co-occurrence (Fig. 2, E and F), suggesting that such vibrational and autofluorescent radiations originate from a common compound. A number of subwavelength-thick accumulations within the mitochondrial network could act as strong scatterers in the CH-stretching region of the Raman vibrational spectrum, such as cardiolipin, cytochrome c, and the double extra-folded lipid membrane typical of their structures (*36*) (see note S3). Consistently, while metabolic enzymes can be found distributed in the cytosol of cells, they accumulate inside the mitochondrial network, where metabolic reactions occur. One can appreciate a considerable positive correlation between TPEF and 2850 cm^−1^ E-CARS in both control (Fig. 2E) and TIS samples (Fig. 2F) (Table 1). This proves a predominantly shared source for the two observed NLO signals. The average M1 colocalization coefficient for control cells amounts to 0.678, which is in agreement with our observation that most of the E-CARS signal spatially corresponds with NAD(P)H and FAD aggregations; thus, one can reasonably associate it with mitochondrial constituents. For TIS cells, M1 is relatively low, amounting to 0.441: Subwavelength-thick lipid aggregations overlap only partially with NAD(P)H and FAD signals on a pixel scale (i.e., 350 nm), suggesting that a portion of the small lipid beads detected are not related to mitochondrial compounds but consist of small cytoplasmic lipid vesicles. This finding is in excellent agreement with the overproduction of lipid droplets in TIS cells, demonstrated in the previous analysis of F-CARS and SRS channels. Accordingly, the averaged M2 colocalization coefficient is 0.630 in control samples and 0.830 in TIS counterparts: TPEF from NAD(P)H and FAD colocalizes more with the E-CARS signal when there is evidence of mitochondrial contraction in TIS cells, giving rise to a higher density of mitochondria-related Raman scatterers able to generate detectable E-CARS radiation. Overall, NLO data suggest that two effects are simultaneously occurring in TIS cells (Fig. 2F): Cellular stress induces subwavelength lipid droplets to accumulate (M1 decreases), while the signal from NAD(P)H and FAD results in dense, bright spots (M2 increases). Therefore, compared to control cells in which M1 and M2 present equal values (*P* = 0.455), TIS cells show diverging M1 and M2 colocalization coefficients (*P* = 4.9 × 10^−5^). While control cancer cells showed a large distribution of signals in both the E-CARS and TPEF channels around the ellipsoidal shapes of nuclei (Fig. 2A), the signals appear as small bright spots sparsely distributed within TIS cells (Fig. 2B). This observation implies that the rearrangements in mitochondrial constituents are linked to the onset of senescence (*37*), as it is further discussed in the following paragraph. To support these findings, we observed MitoTracker-stained HepG2 cells in TIS and control conditions. Although label distribution may be intrinsically affected by the link mechanisms of dye molecules, which is not uniquely related to endogenous fluorescence and molecular vibrations detectable from dense accumulations of scatterers, a rearrangement of mitochondria is clearly observed (figs. S2 and S4), as further detailed in note S2.

NRB-free SRS images of cellular proteins and lipids at 2930 and 2850 cm^−1^ Raman shifts, respectively, could be linearly subtracted to recover the subcellular distribution of pure proteins (*36*, *38*). Therefore, regions primarily of nucleus with minimal contributions of lipids can be localized. These nuclei sites were verified by the presence of a similar spatial distribution between this SRS difference signal and the signal at 2970 cm^−1^ Raman shift, corresponding to the band of deoxyribose CH-stretching vibrations (*38*). This signal was effectively collected via F-CARS, owing to the inherent self-heterodyne amplification given by the NRB. Although the SRS subtraction signal suffers from low quality because it is the result of a pixel-wise subtraction on measured intensity values obtained with two subsequent scans at different Raman modes, impairing a rigorous pixel colocalization analysis with F-CARS maps at 2970 cm^−1^, a qualitative spatial correspondence between these images of nuclei is clear by visual inspection and confirmed by quantitative analyses (see table S1). Last, linear light transmission outlined the cell footprint with respect to the substrate.

The combination of such different ultrafast light-matter interactions and detection methods, co-registered on a pixel-wise scale, was crucial to unveil subcellular chemical maps of label-free human cancer cells (i.e., lipid droplets, mitochondria, and nuclei) by providing enough chemical details for the scope of this study as well as validating the origin of the observed signals through complementary results from different NLO modalities. The use of a multimodal approach enabled us to confirm the robustness of signals, corroborating the presence and location of targeted analytes as well as averting the generation of spurious maps. Thus, quantitative colocalization analyses have been used between NLO channels throughout this study, serving as a validation of image and statistical analysis, as detailed in the following sections. Also, these techniques can be developed under practical conditions and allow us to perform ultrafast chemical imaging (i.e., 1.5 ms per pixel), surpassing other super-resolution microscopy methods (*39*), thus paving the way for future exploitation as easy-to-use screening tools.

### Redistribution of NAD(P)H and FAD via TPEF microscopy is an early-TIS hallmark

**Fig. 3.**
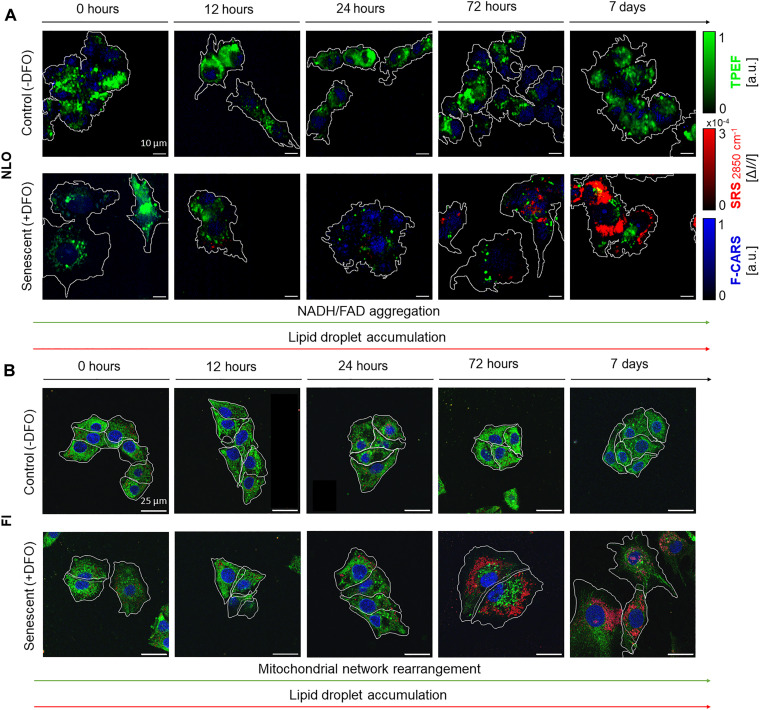
Comparison of label-free multimodal NLO images and labeled confocal microscopy images of HepG2 cells. Both NLO and fluorescence imaging (FI) images were collected over a period ranging from 0 hours to 7 days of culture. The rearrangement of mitochondria is qualitatively evident via NLO microscopy, starting early treatment time. One can appreciate a strong and clear accumulation of lipid vesicles with both NLO and FI microscopy starting 72 hours of treatment. (**A**) In multimodal NLO images, TPEF signals from NAD(P)H and FAD are shown in green, 2850 cm^−1^ SRS signals from lipids are shown in red, and 2970 cm^−1^ F-CARS from deoxyribose in nuclei is shown in blue. (**B**) In confocal microscopy images, lipids are stained with BODIPY (shown in red), mitochondria are stained with MitoTracker (shown in green), and DNA is stained with DAPI (shown in blue).

**Fig. 4.**
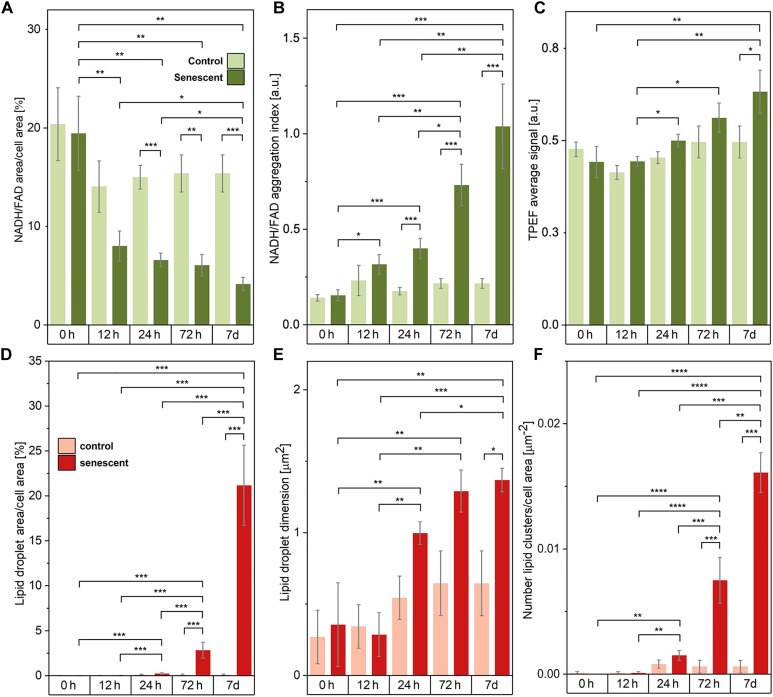
Results of monitoring the onset and progression of traits of TIS cells via multimodal NLO microscopy. Graphs compare control cancer cells with TIS counterparts, over a period ranging from 0 hours to 7 days of culture. (**A**) The graph shows a decrease over treatment time in the percentage of cell area covered by NAD(P)H and FAD signals, as visible in TPEF raw images. Accordingly, (**B**) shows changing values of the mitochondrial aggregation index. (**C**) The average endogenous TPEF signal increases slightly over time of pro-senescence treatment, whereas it maintains a constant value in untreated control cells. (**D**) The percentage of cell area covered by lipid droplets increases over treatment time in TIS cells. (**E**) The area of quasi-single lipid droplets over treatment time enlarges significantly, only in TIS counterparts. (**F**) The number of lipid vesicle clusters inside TIS cells cytoplasms increases significantly over 7 days of therapy. Two-sided Mann-Whitney *U* tests were performed for statistical significance: *0.01 < *P* ≤ 0.05; **P ≤ 0.01; ***P ≤ 0.001; ****P ≤ 0.0001.

TPEF is a linear endogenous fluorescence signal arising from a third-order NLO excitation that scales linearly with the density of NAD(P)H and FAD fluorophores in the focal volume. Its signal can be exploited as an indication of the mitochondrial network location within cells, where metabolic activity is denser (*40*). Under near-infrared excitation (a 780-nm pump beam is used in this study), we collected endogenous TPEF signals by NAD(P)H and FAD ranging between 400 and 600 nm (*41*) (spectrally separated from CARS and E-CARS signals at 650 ± 20 nm). We observed via TPEF microscopy a qualitatively evident (Fig. 3A) and quantitatively significant (Fig. 4, A to C) rearrangement of NAD(P)H and FAD patterns in the cytoplasm of TIS cells, compared to proliferating ones (Fig. 3A). Measurements were carried out at 0 hours, 12 hours, 24 hours, 72 hours, and 7 days after DFO treatment started. The first time point at 0 hours, immediately after DFO administration, accounts for control of any spurious fluorescence given by the pro-senescence drug, as well as temporal control. The use of synchronized controls was intended to exclude any contribution of initial replicative senescence at later culture time points.

The percentage of cell area displaying mitochondrial NAD(P)H and FAD coenzymes (Fig. 4A), defined as the ratio [Area(TPEF)/Area(Cell)]%, shows a steep decrease in treated cancer cells, reaching noticeable differences with respect to controls (i.e., *P* = 1.8 × 10^−4^) starting from 24 hours after treatment start. Such differences are then maintained by cells up to late stages (72 hours, *P* = 10^−3^; 7 days, *P* = 1.8 × 10^−4^). While in normal conditions mitochondrial coenzymes appeared a wide distribution over the cells, occupying on average of 15% of the cell cytoplasm and preferentially gathered around the nuclear region, TIS cells demonstrated contrasting progressions. NAD(P)H and FAD molecules were concentrated and localized, occupying on average 4% of cellular cytoplasm at 7 days of treatment. Such distinct differences are also observed within TIS (with *P* < 0.01) and control groups when comparing the 0-hour treatment time point to the other time points, implying that this observed trend can be associated with a natural change in cell metabolism when adapting to perturbation. Because of cell volume enlargement and flattening associated with the establishment of the TIS phenotype (as reported in the following QPI results section), this index describes metabolic coenzymes’ spatial organization across cells that must be supported by additional observations to conclude that an actual shrinking of the mitochondrial network is occurring in senescent conditions.

Hence, to further describe treatment-induced mitochondrial rearrangements accounting for NAD(P)H and FAD density, we calculated the mitochondrial aggregation index, defined as $\frac{\mathrm{Max}(\mathrm{TPEF})}{[\mathrm{Area}(\mathrm{TPEF})/\mathrm{Area}(\mathrm{Cell})]\%}$, inspired by the work of Haga *et al*. (*42*). This index relates the maximum density of mitochondrial coenzymes, scaling linearly with their endogenous fluorescence peak, with their distribution over the cell area. Differences with respect to controls are observed in senescent cells starting 24 hours of treatment (*P* = 3.3 × 10^−4^), in line with the onset timing of NAD(P)H and FAD spatial rearrangement previously described.

Accordingly, once initiated, the generation of such dense aggregates of coenzymes is maintained over treatment time (72 hours, *P* = 3.3 × 10^−4^ and 7 days, *P* = 7.7 × 10^−4^). Furthermore, as clear from statistically significant differences highlighted in Fig. 4B, this increased localization of NAD(P)H and FAD shows a steep twofold increase in aggregation index within TIS groups from earlier (12 hours, 24 hours) to later stages (72 hours, 7 days) of treatment.

Overall, these quantitative metrics describing NAD(P)H and FAD increased spatial confinement unveil peculiar dynamics in unperturbed mitochondria early after human cancer cells receive pro-senescence stimuli. In excellent agreement with these considerations, the condensation of mitochondria into budding-like shapes was previously described by observing labeled mitochondria via laser scanning cytometry: It occurs as an upstream event of cytochrome c release after anticancer drug treatment of fibrosarcoma cancer cells (*42*). Mitochondrial morphology and distribution, regulated by fusion and fission events, play a crucial role in both senescence and apoptosis cell paths (*43*). Accumulation of dysfunctional mitochondria, namely, a condition in which regulation of their homeostasis, production of specific metabolites, membrane potential, and reactive oxygen species (ROS) generation are altered (*2*), was reported also via immunostaining confocal microscopy and gel electrophoresis, labeled and destructive techniques, respectively, in early senescent fibroblast cells (*44*).

Regardless of its spatial distribution in cells, the average intensity of TPEF provides insights into the metabolic condition of cells as it relates to the average amount of NAD(P)H and FAD produced (Fig. 4C). Over the treatment time, the average TPEF signal from cancer cells showed a slight increase, reaching a significant difference with respect to untreated cells at 7 days of treatment (*P* = 0.02). TIS cells exhibited an appreciable 40% growth of NAD(P)H and FAD signals from early treatment time points to later ones. Earlier studies associated the boost of TPEF signal with photodamage induced by near-infrared radiation, as sequential scans produced progressively higher endogenous fluorescence levels (*45*). Here, as we do not vary the radiation exposure time per field of view (FOV) among sample conditions or sample time points, we exclude photodamage-related causes, attributing the rise of TPEF signal intensity over treatment time to biochemical evolutions taking place at the cellular level. This is aligned with the results of Bennett *et al.* (*46*), who observed equivalent signal behavior when monitoring the viability of pancreatic islets via TPEF, and associated autofluorescence changes with changes in NAD(P)H concentration. Here, we consider the progressively increasing average TPEF as an indication of senescent phenotype progression, related to the overexpression of NAD(P)H and FAD coenzymes. For any index considered, control measurements at 0 hours do not show any statistically significant difference between TIS and untreated cells, as expected, proving our temporal evolution analysis trustworthy and confirming the absence of TPEF spurious signal from administrated drug molecules.

To compare the information provided by NLO imaging approaches to the one given by standard methods under equivalent experimental conditions, we performed confocal fluorescence imaging (FI) on labeled mitochondria in TIS HepG2 cells and controls (Fig. 3B). A qualitative morphological rearrangement of mitochondrial networks was observed over treatment time, supporting the quantitative findings highlighted through TPEF microscopy. Nevertheless, differences between control and treated samples were barely visible after 72 hours of treatment (see note S2), thus losing the advantage of an early detection of TIS phenotype reprogramming. On top of that, direct and precise quantification of mitochondrial changes is not rigorously feasible through such perturbative methods, in which detected signals strongly depend on label molecules and their penetration and retainment, instead of targeted species in their pristine form. Here, we show labeled counterparts to provide a qualitative picture of the analyte via standard tools, to be used for reference and corroboration of results.

### Lipid accumulation detected via SRS microscopy is a trait of established TIS

SRS microscopy tuned at 2850 cm^−1^ Raman shift probes CH_2_-stretching vibrations of dense lipid deposits in the cytoplasm of cancer cells, without any NRB, typical of CARS, nor relevant fluorescence backgrounds. The collected images showed a clear overproduction of lipid vesicles at advanced stages of treatment, whereas no trace of this phenomenon was present in proliferating cancer cells (Fig. 3A). To assess these observations quantitatively and statistically, we measured control and TIS cells at 0 hours, 12 hours, 24 hours, 72 hours, and 7 days after DFO treatment started. Similar to the TPEF analysis, the first time point served as temporal control and control of any spurious signals from the drug used for senescence induction. To exclude our analyses from any contribution of replicative senescence during later culture time, we used temporally synchronized controls.

The percentage amount of cell area featuring 2850 cm^−1^ SRS signal from cytoplasmic lipid vesicles, namely, $\left[\frac{\mathrm{Area}(\mathrm{SRS})}{\mathrm{Area}(\mathrm{Cell})}\right]\%$, is negligible until 24 hours of treatment (Fig. 4D), as also clearly visible qualitatively in Fig. 3A. It amounts to 2.84% at 72 hours of pro-senescence treatment, reaching a precipitous production peak of 21.17% at 7 days. The onset of statistically significant differences between controls and TIS cells occurs at late time points, featuring *P* = 1.6 × 10^−4^ and *P* = 4 × 10^−4^ at 72 hours and 7 days of treatment, respectively. Strong differences are present between these TIS states and the earlier ones at 0, 12, and 24 hours (i.e., *P* < 10^−3^). To further describe lipid droplet overexpression in TIS cancer cells, we monitored the dimension of quasi-single vesicles, along with the number of clusters of vesicles that cells accumulated in the cytosol over treatment time (see the “Processing of multimodal NLO images” section in Materials and Methods). As for the area of lipid droplets, initially, it was 0.4 μm^2^ on average in senescent cells, comparable to controls, and then it significantly increased during the monitored culture period (Fig. 4E). At 7 days, TIS samples displayed more than twofold bigger lipid droplets of circa 1.4 μm^2^ compared to controls (*P* = 1.6 × 10^−2^). Such an overproduction of progressively bigger lipid vesicles is not concentrated in a single mass in the cell cytoplasm but distributed in multiple lipid clusters. By referring quantitatively to this aspect, the number of lipid clusters per cell area (Fig. 4F) significantly grows by one order of magnitude with respect to controls at 72 hours of treatment (*P* = 3.6 × 10^−4^), and up to two orders of magnitude at 7 days (*P* = 1.1 × 10^−4^). Overall, measurement sets at 0 hours do not show any significant difference between TIS and untreated cells, proving the absence of spurious signals from drug molecules, as well as the reliability of the temporal analysis of SRS signals from lipids.

Overall, our findings demonstrate notable lipid accumulation in TIS human cancer cells with a quantitative measure through noninvasive NLO SRS imaging. These results align with highlights of the up-regulation of lipid processing and metabolism in TIS cells via standard and invasive techniques, such as colorimetric assays (e.g., Oil Red O), which rely on label-related signals, and Western blot gene expression analysis, which implies sample destruction (*47*). Flor *et al*. (*10*) proved via a combination of validated assays that proteins and pathways involved in lipid biosynthesis, binding, import, and storage are prominent in human TIS cancer cells treated with etoposide, doxorubicin, and camptothecin chemotherapeutic agents, leading to an overall accumulation of lipids. Moreover, label-free linear optical SR in aged human fibroblast cells pointed out an analogous up-regulation of lipids at different Raman bands, including the 2850 cm^−1^ Raman mode used in this study (*13*). To thoroughly compare our findings to standard ones with consistent experimental conditions, we analyzed BODIPY-labeled lipid vesicles in stained HepG2 cells through confocal fluorescence imaging, over 7 days of treatment. A qualitative overproduction of lipids was observed, confirming the soundness of the quantitative observation derived here from raw SRS channel analysis (see note S2). Because of the all-optical nonperturbing nature of our advanced tools, one can unlock a framework for early detection and profiling of TIS onset and evolution in human tumor cells, when in their original morpho-molecular state, by using a simple, fast, and intrinsically quantitative approach that is also unbiased by any foreign label–related artifact.

### QPI identifies morphological and physiological changes in TIS cells

**Fig. 5.**
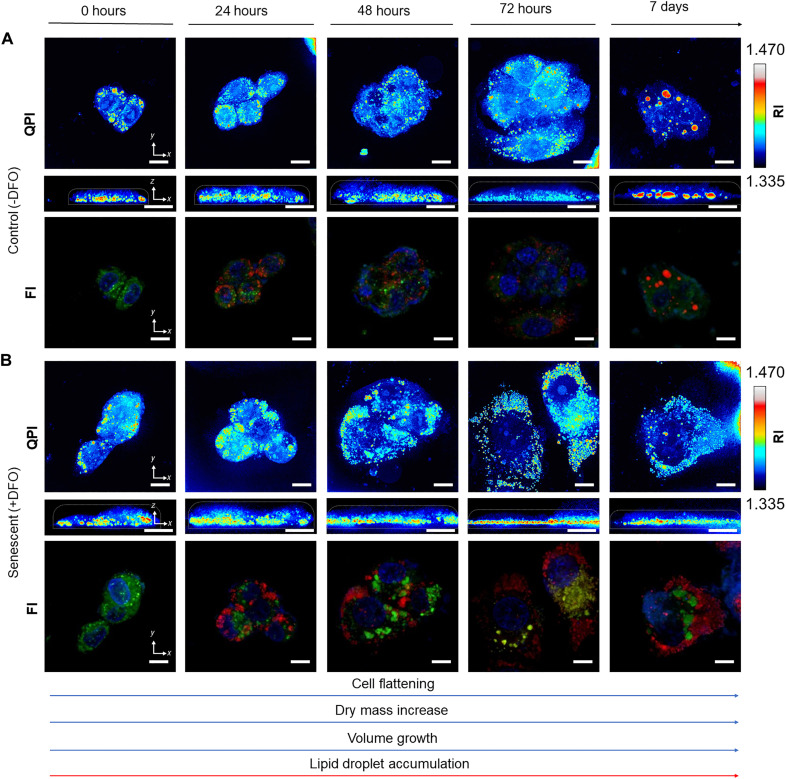
Representative images by label-free QPI and correlated fluorescence imaging of control and senescent HepG2 cells. The images of both DFO-treated and untreated cells were collected over a period ranging from 0 hours to 7 days of culture. (**A**) Control, untreated cells were kept in proliferating conditions for 7 days. (**B**) Senescent cells were treated with DFO for 7 days for senescence induction. Evident accumulation of lipid vesicles is observed as high RI intensity regions over the progression of DFO treatment, while minimal lipid vesicles are present in the control throughout the monitoring. Such observation in QPI corresponds with fluorescence counterparts with cells stained with BODIPY, MitoTracker, and Hoechst to localize lipid droplets (shown in red), mitochondria (shown in green), and nucleus (shown in blue), respectively. Scale bars, 10 μm.

QPI profiles cell structures at high resolution by quantifying phase delay at the wavefront due to the specimen that we used for visualizing TIS and untreated HepG2 cells. We monitored early and late TIS by using QPI and found both physiological and morphological changes, unique to TIS phenotype (Fig 5B), when compared to untreated control cells (Fig. 5A). Cells were imaged at 0 hours, 24 hours, 48 hours, 72 hours, and 7 days of treatment with QPI, followed by fluorescence staining and imaging of the same cells to localize their structures (Fig. 5). Lipid droplets were clearly identified as bright regions in QPI images that match with the fluorescent counterpart of the same cells. Compared to the control cells (Fig. 5A) with only few lipid droplets observed at all time points, TIS cells (Fig. 5B) exhibited several overproduced lipid regions. Lipid droplets at early TIS are highly clustered and spotted within the cell, and late-TIS measurements showed lipid droplets covering a larger area of the cell and enclosing enlarged cytoplasm. This QPI result is in agreement with the abovementioned F-CARS, E-CARS, and SRS measurements that demonstrated clear lipid accumulation in senescent cells over the progression of the treatment. Also, the overall size of induced senescent cells constantly increased: A substantial increase was observed in late treatment stages (72 hours and 7 days of DFO treatment) so that a single cell encompassed the image plane. On the other hand, untreated cells remained similar in size. Changes in cell thickness were observed as well in late-stage TIS, with cells exhibiting a flatter morphology compared to controls, which maintained a similar thickness throughout the monitoring period. These morphological changes identified by QPI in label-free conditions are in agreement with previously reported studies, describing enlarged and flattened morphology of senescent cancer cells (*48*, *49*).

**Fig. 6.**
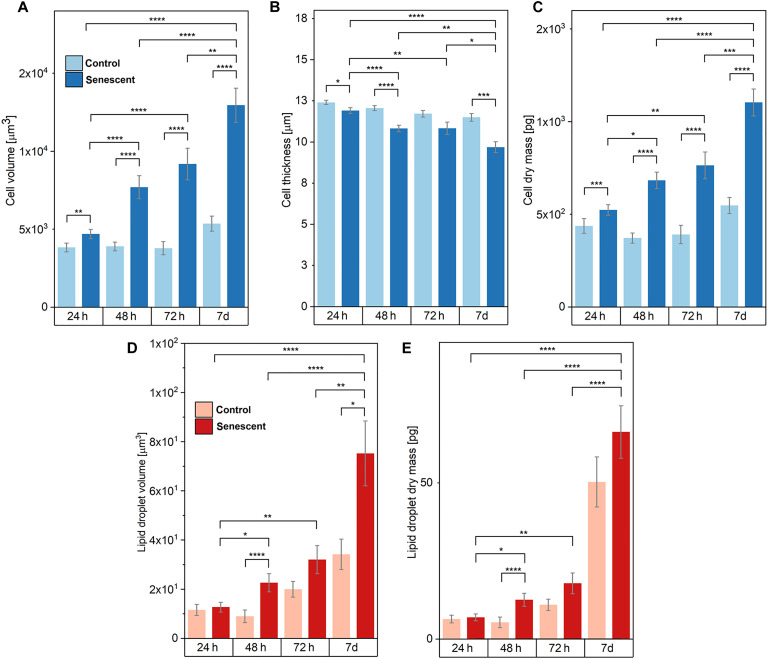
Quantitative analysis of control and senescent HepG2 cells over the duration of monitoring from 0 hours to 7 days of culture via QPI. (**A**) The average volume of DFO-treated senescent cells increased over time, whereas that of control ones remained constant. (**B**) The average cell thickness decreased over the DFO treatment. (**C**) The average cell dry mass of DFO-treated cells increased over the monitoring duration, while that of untreated cells remained constant. (**D**) The average volume of lipid droplets in a single cell, calculated by the number of voxels occupied by lipid droplets within the cell, showed a gradual increase in both control and senescent cells, yet a significant increase was observed in senescent cells at late TIS. (**E**) The average dry mass of lipid droplets in a single cell increased over the DFO treatment time with a significant increase in day 7 of the treatment. Two-sided Mann-Whitney *U* test was performed for statistical significance: *0.01 < *P* ≤ 0.05; **P ≤ 0.01; ***P ≤ 0.001; ****P ≤ 0.0001.

Such physiological and morphological observations from visual inspections were quantitatively analyzed with respect to volume, dry mass, and thickness, based on the RI distribution (Fig. 6). The measured RI of senescent cells yielded an overall increase in both volume (Fig. 6A) and dry mass (Fig. 6C), compared to the control cells that remained constant throughout the monitoring. Compared to an early-TIS stage, a marked difference between control and senescent cells was observed in both volume and dry mass with a statistical significance of *P* < 10^−4^. Also, senescent cells at early and late stages showed clear statistical difference (i.e., *P* < 10^−4^). Additionally, the average changes in cell thickness could be assessed from the 3D RI distribution. Although both DFO-treated and untreated cells present a gradual decrease in thickness over time, the senescent cells, specifically in a late-TIS stage, exhibited flattened morphology with a greater decrease in thickness, featured by strong statistical significance (i.e., *P* < 10^−4^), compared to the measurement at 24 hours of treatment (Fig. 6B). Such flattened cell morphology is clear in the cross-sectional projection of cellular RI distributions (Fig. 5).

Similar findings were observed in lipid droplet distribution. Lipid droplets can be visibly inspected within RI maps of cells, marked by high intensity regions, since lipid droplets have relatively high RI values compared to the rest of most cell compartments (*50*, *51*), as well as verified with correlated fluorescence counterparts. The average lipid volume (Fig. 6D) and dry mass (Fig. 6E) of senescent cells constantly increased throughout the treatment, with a noticeable increment on day 7 marked by statistical significance (i.e., *P* < 10^−4^), compared to 24 hours of treatment. On the other hand, the average volume and dry mass of lipid droplets of control cells did not change significantly during early TIS. Nevertheless, at later time points of untreated cells, a gradual increase in lipid volume was observed, together with a rapid surge in lipid dry mass (7 days). Such an observation indicates that although both exhibit the formation of lipid vesicles, there is a distinction in the density between lipid droplets formed in untreated and DFO-treated HepG2 cells, specifically at a late-TIS stage. Senescent cells have a relatively constant lipid dry mass–to–volume ratio throughout the duration of DFO treatment, while untreated proliferating cancer cells form lipid droplets that gradually become denser over time.

### TIS exhibits consistent NLO traits in radiation therapy conditions

To support the generalizability of TIS manifestation as observed through the proposed all-optical label-free techniques, we monitored HepG2 cultures treated with radiation therapy, the current gold standard for anticancer treatment, widely practiced in the clinics (*52*). Cultures were monitored for a 20-day follow-up period. To quantitatively and statistically evaluate the trends of TIS markers presented by DFO treatment, we measured radio-TIS cells at 5, 10, and 20 days after exposure to 10-Gy γ radiation. Being radiotherapy not designed as a pro-senescence treatment for in vitro modeling of TIS as the DFO-driven method used for this study, the specific timeline of cell response to consider extends to 2 to 3 weeks (*53*). Untreated HepG2 cells, observed at the initial 5-day time point, served as a sole control, as no statistically significant variations of TIS markers were present in these counterparts over time (Fig. 4). NLO imaging was used for this part of the study; its associated TPEF and SRS optical markers were evaluated for early-stage TIS detection.

**Fig. 7.**
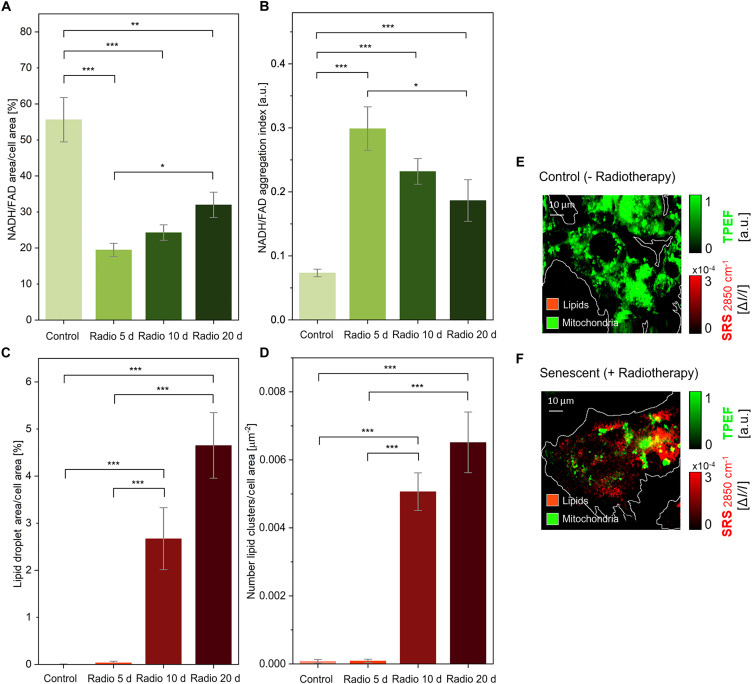
Quantitative analysis of control and radiotherapy-induced senescent HepG2 cells over 20 days of follow-up. (**A**) Decrease in the percentage of cell area covered by NAD(P)H and FAD signals in TIS cells, as visible in TPEF raw images. Later stages exhibit a slight increase in this index. (**B**) The graph shows increased values of the mitochondrial aggregation index in TIS cells. Accordingly, the index increases slightly over follow-up measurements. The percentage of cell area covered by lipid droplets increases steeply over treatment time in TIS cells (**C**), as well as the number of deposited lipid vesicle clusters (**D**). Qualitatively, the distribution of mitochondria-associated signal (green, TPEF) and lipid-associated signal (red, SRS) clearly varies, compared to untreated HepG2 cells (**E**) and cells that underwent radiation therapy, as observed after 20 days through multimodal NLO imaging (**F**). Two-sided Mann-Whitney *U* test was performed for statistical significance: *0.01 < *P* ≤ 0.05; **P ≤ 0.01; ***P ≤ 0.001.

Notably, both the rearrangement of NAD(P)H and FAD in cell cytoplasm and the abrupt overproduction of lipid vesicles, as probed via TPEF (Fig. 7E) and SRS (Fig. 7F) microscopy, respectively, appeared as consistent trends with respect to the ones uncovered by DFO-mediated TIS models. Also, the chronological timeline of events taking place in TIS-committed hepatic cancer cells, as described through TPEF and SRS, was maintained. Specifically, hepatic cancer cells display an early-stage aggregation of mitochondrial co-enzymes, with strongly significant differences (i.e., *P* < 10^−3^) with respect to the controls at only 5 days after radiation exposure (Fig. 7, A and B). The spatial distribution of NAD(P)H and FAD barely changed in radio-TIS over the follow-up, consistent with the observation from the DFO model (Fig. 4, A and B). On the other hand, SRS signals described a later cellular response. The production of lipid droplets aggregated in progressively more numerous clusters became clearly visible 10 days after radiotherapy, with a steep difference in the percentage of cell area showing lipid accumulations and in the number of lipid clusters deposited (*P* = 1.3 × 10^−4^) compared to the controls. No evidence of such lipid content increase was visible at earlier stages, with a comparable SRS signal distribution at the 2850 cm^−1^ Raman shift of CH_2_ stretching for radio-TIS and controls at 5 days (refer to fig. S7 for additional quantitative indexes of TIS derived from multimodal NLO image analyses). Also, from a qualitative standpoint, TIS cancer cells in radiation therapy conditions appeared visibly distinct, with more largely distributed TPEF signals in normally proliferating cultures (Fig. 7E) and evident 2850 cm^−1^ SRS signal detected in senescent phenotypes (Fig. 7F). To further investigate the generalizability of our results, we tested doxorubicin, one of the most common chemotherapeutic agents currently used in clinical practice, with two different human cancer cell lines (i.e., HepG2 and TPC-1). We could reveal TIS manifestation with comparable early NLO traits (refer to note S4 for multimodal NLO images of 7-day doxorubicin-doped HepG2 and TPC-1 human cancer cells).

## DISCUSSION

Here, we used multimodal vibrational and multiphoton microscopy coupled with interferometric phase imaging and retrieved both chemical and morphological key cell information, respectively. We could describe the early morpho-molecular manifestation of TIS in human cancer cells, a phenotype potentially driving tumor dormancy and recurrence, which has been poorly investigated so far in its pristine and unperturbed original form.

These advanced methods allowed us to quantitatively, rapidly, and noninvasively distinguish the critical senescent phenotype in human tumor cultures that underwent senescence induction (Fig. 2). Exploiting the intrinsic nature of these light-matter interactions enabling quantitative profiling of biochemical and morphological cell features, we provided a comprehensive description of TIS, including mitochondrial network rearrangement, lipid overproduction, cell flattening, and cell enlargement. We managed to reveal the onset timeline of such hallmarks and provide insight into their chronological evolution with statistical significance, by analyzing a fairly large number of human cells and overcoming the high biological variability that typically governs these mechanisms. Our results revealed diverse TIS features, falling into either the molecular or morphological cellular profile, which can consistently be correlated with observations among modalities: We could demonstrate consistent signals in cases of a shared origin of the target (e.g., lipid beads in the cytoplasm revealed either via CARS/SRS or via QPI) and in terms of chronological onset timelines (both morphological and molecular rearrangements start being significant at 24 hours after therapy starts). Compared to control proliferating cancer cells, TIS cancer cells exhibited a 3-fold more shrunk pattern of NAD(P)H and FAD coenzymes in the mitochondrial network (Fig. 4, A to C), and a 20-fold higher accumulation of progressively bigger lipid droplets gathered in cytoplasmic clusters (Fig. 4, D to F), together with qualitatively more irregular and larger nuclei. Morphologically, TIS cells appeared flattened to 75% of the initial thickness (Fig. 6B) and enlarged, featuring a threefold increased volume (Fig. 6A) and more than doubled dry mass (Fig. 6C), compared to untreated proliferating counterparts. These quantitative findings, all of which were observed in label-free conditions, not only agree with qualitative senescence markers that have previously been identified via standard, slow, invasive, and/or destructive techniques but also endorse accurate, early identification of TIS phenotypes by providing quantitative measures based on morpho-molecular properties of TIS cells. This approach can potentially be applied to multiple arrays of TIS study in clarifying its involvement in anticancer therapy that will ultimately support advancing current therapeutic practices.

By analyzing a large cancer cell dataset, using recent TIS biological models thoroughly validated elsewhere (*25*, *54*), we revealed notably reproducible quantitative morpho-chemical traits featured by TIS human cancer cells over early reprogramming stages. As the TIS markers here presented are visible via NLO microscopy and QPI, the community would benefit from the advantages of these easy-to-use, label-free, rapid, and quantitative optical technologies to foster further TIS exploration in cancer therapy screening, when using both in vitro cell culture models and patient-derived cell cultures. Even with a limited amount of cancer cells from patients, one can quantitatively assess the efficacy of treatment through this morpho-molecular imaging approach, advancing therapy screening by using fast and high-content quantitative microscopy directly on nonperturbed samples, without requiring any manipulation, destruction, or labels.

For wider exploitation in diverse real-world scenarios, the current study should be further generalized by testing varied human tumor cell lines and investigating their TIS expression under diverse anticancer stimuli. Our experiments on radiotherapy-induced senescence confirmed the consistency of TIS-associated NLO signals as demonstrated here, thus supporting the generalizability of our approach to foster future research and applications. Additionally, we observed comparable TIS label-free traits in different human cancer cell lines treated with standard doxorubicin chemotherapy (note S4). As we target to uncover the major manifestations of senescence at an earlier phenotype commitment stage, such as mitochondrial dysfunction, morphological enlargement and flattening, and alterations of the lipid profile, distinct phenotypes under different treatments may undergo peculiar reprogramming pathways toward senescence, with specific timelines for the manifestation of early-TIS markers, which should be further explored.

To these aims, our findings provide fundamental knowledge about original TIS early dynamics in human cancer cells. By exploiting diverse label-free, quantitative, rapid, and easy-to-use technology, we describe the comprehensive morpho-molecular profile of TIS over early reprogramming while dismissing the potential contribution of any spurious artifact due to labeling and/or manipulation of cells as typical in standard analyses. This study is a crucial step toward promptly evaluating TIS, a critical phenotype in anticancer therapy screening. We envision further application of our methods and exploitation of our findings in TIS studies to improve current screening protocols for anticancer therapy.

## MATERIALS AND METHODS

### Cell culture and treatment

HepG2 cells were purchased from the American Type Culture Collection (ATCC; Manassas, VA, USA; ATCC number: HB-8065) and maintained in Dulbecco’s modified Eagle’s medium (DMEM) (Gibco) supplemented with 10% fetal bovine serum (FBS) (Gibco) at 37°C and 5% CO_2_. To trigger the development of senescence-associated features, DFO (Merck) was diluted in distilled water at the final concentration of 100 μM in the culture medium. Cells were exposed to DFO starting 24 hours after plating and cultured in its presence for 12 hours, 24 hours, 72 hours, or 7 days.

### Confocal immunofluorescence microscopy

For immunofluorescence analysis, cells were plated on 35-nm glass-bottom dishes. Mitochondria were stained in live cells with MitoTracker Deep Red FM (Invitrogen) diluted at the final concentration of 100 nM in the culture medium. Cells were incubated with the probe at 37°C and 5% CO_2_ for 45 min. After the incubation period, cells were gently washed with phosphate-buffered saline (PBS) (Lonza) and fixed with 4% PFA for 10 min. Fixed cells were gently washed with PBS, and the glass-bottom dishes were mounted on microscope slides with DAPI (4′,6-diamidino-2-phenylindole)–containing antifade reagent (ProLong Gold, Life Technologies) and dried overnight. Lipids were labeled through BODIPY fluorescence staining. Confocal microscopy was performed with a Leica TCS SP8 X confocal laser scanning microscope (Leica Microsystems GmbH, Mannheim, Germany).

### Multimodal NLO microscopy

For NLO microscopy, cells were cultured on 22 mm × 22 mm × 0.17 mm quartz slides (UQG Optics, UK) at a density of 320,000 cells/ml. Once the desired culture time was reached, cells were fixed in 4% PFA for 10 min and stored at 4°C. Before measurements, each quartz slide was mounted upside-down and sealed with a second 25 mm × 50 mm × 0.17 mm quartz slide (UQG Optics, UK), with cells placed in between the two slides. For QPI, cells were cultured on 50-mm glass-bottom petri dishes (MatTek) at a density of 160,000 cells/ml. Cells were fixed at the desired time points with the same procedure described for NLO microscopy samples. The temporal control at 0 hours refers to cells fixed right after the introduction of DFO.

We used a homebuilt multimodal optical microscope featuring seven different NLO modalities (fig. S6), along with linear transmission light: forward-detected stimulated Raman scattering (SRS), forward and epi-detected coherent anti-Stokes Raman scattering (CARS and E-CARS), two-photon excited fluorescence (TPEF and E-TPEF), and second-harmonic generation (SHG and E-SHG). This setup has been described in detail elsewhere (*55*–*57*). Briefly, a compact erbium-fiber multibranch laser source (FemtoFiber Pro, Toptica Photonics, Germany) delivers 1560-nm pulses at 40-MHz repetition rate with <100-fs duration. One branch is frequency-doubled to deliver narrowband 780-nm pump pulses, whereas a second branch is spectrally broadened and then frequency-doubled to allow the generation of tunable picosecond Stokes pulses in the range of 950 to 1050 nm. Both the resulting pump and Stokes pulses feature ≈1-ps duration, and their frequency detuning matches with the CH-stretching region of the Raman vibrational spectrum (2800 to 3100 cm^−1^). Average laser powers at the sample plane were kept constant at 7.5 mW for the pump and 0.5 mW for the Stokes throughout the study. The inverted-configuration microscopy unit includes high numerical aperture (NA) objectives that ensure subcellular spatial resolution [~280-nm lateral resolution, ~300-nm axial resolution, as for the Rayleigh criterion applied to NLO imaging (*58*)]: a water-immersion 100× 1.25 NA 0.25-mm working distance (WD) illumination objective (C-Apochromat, Carl Zeiss, Germany) and an oil-immersion 40× 1.35 NA 0.19-mm WD (CFI Super Fluor, Nikon, Japan) collection objective. We provide an experimental evaluation of the maximum lateral resolution achievable by the system in fig. S10, amounting to 267 ± 17 nm. The *x*-*y*-*z* motorized sample stage (Standa, Lithuania, and Mad City Labs Inc., USA) can scan very large areas (up to 50 mm × 50 mm) while keeping the sample always in focus. A multichannel detection scheme allows performing three imaging techniques simultaneously: A lock-in amplifier (Zurich Instruments, Switzerland) demodulates forward SRS at 1 MHz [in the form of stimulated Raman gain (SRG) at Stokes photon energies], with an acousto-optic modulator (AOM) modulating the pump beam. SRG pixel intensity values are reported as actual differential signals {i.e., to ease reproducibility of results, namely, computing the ratio of the signal extracted via lock-in amplification [output in volts root mean squared (VRMS)] normalized over the Stokes intensity at the balanced photodiode (output in volts)}. A photo-multiplier tube (PMT) (Hamamatsu Photonics, Japan) detects forward CARS, TPEF, or SHG according to the optical filtering at its inlet. Similarly, a backward PMT is used for E-CARS, E-TPEF, or E-SHG. All cell images in the present study have a dimension of 105 μm × 105 μm, 300 pixels × 300 pixels (i.e., a pixel size of 350 nm × 350 nm; hence, the lowest lateral resolution is 700 nm for the Nyquist sampling theorem). A 1.5-ms pixel dwell time is used for multichannel image acquisition.

### Processing of multimodal NLO images

Images were processed via the Fiji-ImageJ software for image analysis and Python for the colocalization study. Both dark and bright 1-pixel outliers in every channel of multimodal images were median-filtered to correct the extreme pixel values given by cosmic rays. An automated circular shift of image columns is used to compensate for distortion effects due to the serpentine-like motion of the motorized sample stage. As for the images collected from SRS microscopy, the SRG signal extracted via lock-in amplification (Δ*I*) was normalized over the linear transmission of the Stokes beam through the sample (*I*) to obtain the target normalized Δ*I*/*I* SRG signals. The linear transmission of the Stokes is used to differentiate cells from the substrate and outline the cell area, as cells appear as relatively dark regions due to a drop in laser transmission. As for mitochondrial NAD(P)H and FAD metrics quantified from raw TPEF images acquired with constant integration time and laser power throughout this study, a universal threshold of 0.25 arbitrary unit (a.u.) was set to distinguish the endogenous fluorescence signal from the diffused background signal that presented outside of cells. Similarly, for lipid metrics derived from raw SRS images acquired with constant integration time and laser power, a universal threshold of 2.2 × 10^−4^ Δ*I*/*I* was used to separate dense vesicles from low-density diffused cytoplasmic lipids and background. Quasi-single lipid droplets were identified through the Analyze Particles tool in Fiji-ImageJ (*59*), using as filter arguments a particle size in the range 5-infinite [pixel^2^], and a circularity in the range of 0.3 to 1, so to isolate lipid beads with moderate-to-high circularity that can reasonably correspond to quasi-single vesicles. Without any constraint on circularity, the same tool was used to enumerate lipid clusters, gathered in the cytoplasm of cells with varied and irregular shapes. To calculate the maximum value of TPEF displayed by cells in an image, we considered the average value of the 10% highest pixel values. This procedure, together with the aforementioned median filtering of outlier pixels, allowed us to avoid spurious TPEF pixel values and obtain a metric, which effectively scales with the maximum density of fluorescent scatterers in the FOV. As the aggregation index should feature at the numerator a value scaling with the density of target molecules regardless of their spatial distribution, which is taken into account in the denominator instead, we used TPEF maxima rather than mean or SD values.

### NLO channels colocalization analysis

In colocalization analyses (between SRS and F-CARS channels at 2850 cm^−1^, and between TPEF and 2850 cm^−1^ E-CARS channels), we calculated the PCC and Manders overlap coefficients (M1, M2) (*33*, *60*) to quantify the degree of pixel-wise signal co-occurrence between co-registered nonlinear modalities (Fig. 2, C to F). Conventionally referring to the signal on the *x* axis of colocalization plots as channel 1 and the one on the *y* axis as channel 2, M1 is the fraction of channel 1 and channel 2 co-occurring pixel frequencies (top-right quadrant), over the total pixel frequencies of channel 1 (top- and bottom-right quadrants); M2 is the fraction of channel 1 and channel 2 co-occurring pixel frequencies (top-right quadrant), over the total pixel frequencies of channel 2 (top-right and top-left quadrants). A two-sided Student’s *t* test was performed to assess differences between M1 and M2 in TIS cells and proliferating counterparts (*n* = 10 samples each). The Costes method (*61*) was used as a control to test the statistical significance of our results against colocalization measurements performed on randomly rearranged images, in which the distributions of the signals in the two channels are unrelated and should yield random correlation.

### Statistical analysis of NLO signals

For each time point (i.e., 0 hours, 12 hours, 24 hours, 72 hours, and 7 days) considered in our study (Fig. 3), we analyzed 10 different culture plates, divided equally into 5 controls and 5 DFO-treated counterparts. For each plate, two 105 μm × 105 μm FOVs were acquired, chosen randomly on the culture substrate, and containing 10 cells on average. Hence, for each condition (control versus senescent) at each one of the five time points, we considered a population of 100 HepG2 cells. This fairly large number of cells allowed us to account for biological batch-to-batch variability. The statistical significances between control and TIS cells at each time point, as well as within TIS and control groups, were measured through a nonparametric two-sided Mann-Whitney *U* test, considering a significance level of 0.05.

### Quantitative phase imaging

The quantitative phase images of cells were obtained with a commercial tomographic phase microscope, HT-2H, with a lateral and axial resolution of ~110 nm and ~356 nm, respectively (Tomocube, South Korea). All QPI images of cells have a dimension of 80.470 μm × 80.470 μm × 39.666 μm (848 pixels × 848 pixels × 210 pixels). For each QPI image, corresponding fluorescence images were also captured to localize subcellular structures. To confirm the localization of the nucleus, lipid droplets, and mitochondria, live cells were stained with Hoechst (Thermo Fisher Scientific), BODIPY (Invitrogen), and MitoTracker Deep Red FM (Invitrogen), respectively. Stained cells were washed with PBS (Thermo Fisher Scientific) and then fixed with 4% PFA.

### Processing of QPI images

QPI images of cells were mapped based on the RI values of cell components and analyzed using TomoStudio software (Tomocube, South Korea). The cell volume was calculated by segmenting cells based on the RI of PBS background (RI = 1.3342) and integrating the number of voxels that occupy each cell. We applied 0.19 and 0.135 ml/g for the RI increment (*dn*/*dc*) of the cell and lipid droplets, respectively, which are well-established values by previous reports (*62*). The cell dry mass was calculated by the surface integral of the optical phase shift due to the cell, as detailed in earlier reports (*23*), $\mathrm{DM}\approx \int_{A}\phi (x,y)dxdy$, where DM is the cell dry mass, λ is the illumination wavelength, ϕ is the average phase optical shift, and *A* is the surface area. Accordingly, the cell volume and cell thickness were estimated based on the 3D holographic measurement of cells. For lipid droplets, the range of RI, 1.4 ≤ *n* ≤ 1.46, was specified to distinguish the cellular regions occupied by aggregated lipid vesicles within QPI images, facilitated by their relatively high RI, compared to the other cell organelles and structures, which correspond with matching BODIPY-stained images. The identified lipid region is estimated by the number of voxels indicative of lipid droplets within each cell, resulting in the calculation of lipid volume and dry mass. QPI images of cells and lipid droplets were processed and segmented using ImageJ and MATLAB (R2018b), and cell thickness was calculated from the segmentation.

### Statistical analysis of QPI signals

For each time point (i.e., 0 hours, 24 hours, 48 hours, 72 hours, and 7 days) considered in our study (Figs. 4 and 5), we analyzed four different culture dishes, divided equally into two controls and two DFO-treated counterparts. Within every culture dish, approximately 75 cells were randomly selected for imaging and an average of 25 QPI images were collected. Therefore, our analysis addressed biological batch-to-batch variability based on a total population of approximately 150 HepG2 cells, measured from two distinct culture dishes, for each condition (control versus senescent) at five different time points. The statistical significances between control and TIS cells, and within TIS and control groups, for all time points, were calculated in the same manner as conducted for NLO signal analysis, by using a nonparametric two-sided Mann-Whitney *U* test with a significance level of 0.05.
