# Supplementary material for: Noninvasive morpho-molecular imaging reveals early therapy-induced senescence in human cancer cells
Source: Sci Adv. 2023 Sep 13;9(37):eadg6231. doi: 10.1126/sciadv.adg6231 (PMC10881071; doi:10.1126/sciadv.adg6231)
Supplement: Supplementary file 1 — Supplementary Text Figs. S1 to S10 Table S1 [file sciadv.adg6231_sm.pdf]

Science Advances
AAAS

Supplementary Materials for

Noninvasive morpho-molecular imaging reveals early therapy-induced senescence in human cancer cells

Arianna Bresci *et al.*

Corresponding author: Dario Polli, dario.polli@polimi.it; Ishan Barman, ibarman@jhu.edu;
Italia Bongarzone, italia.bongarzone@istitutotumori.mi.it

Sci. Adv. **9**, eadg6231 (2023)
DOI: 10.1126/sciadv.adg6231

This PDF file includes:

Supplementary Text
Figs. S1 to S10
Table S1

Supplementary Text

Supplementary Note 1: β-galactosidase activity and yH2AX foci in DFO-doped senescent HepG2 cell models

To assess the establishment of senescence in HepG2 cells after DFO treatment, we stained them for β-galactosidase (β-gal) activity. To date, β-gal activity detected at pH 6.0 is the most widely accepted biomarker for senescent cells in culture and mammalian tissues: it is low or non-detectable at pH 6.0 in non-senescent cells and it increases significantly when cells become senescent. We stained the cells with two markers for the detection of β-galactosidase: the Cell Signaling SA-β-gal Staining Kit and the Invitrogen Green Biotracker 519 β-gal dye. The SA-β-gal Staining Kit (first row of Fig. S1A) allows the visualization of β-gal activity in brightfield (blue), while Biotracker 519 (second row of Fig. S1A) is a fluorescent dye (green). In addition, we stained the cells for phosphorylated H2A histone family member X (γ-H2AX) foci. γ-H2AX foci are a common marker for senescence as they are directly correlated to DNA damage, which is a global feature of cellular senescence. Moreover, senescent cells can exhibit γ-H2AX foci even without the presence of a DNA-damaging agent.

Supplementary Note 2: Confocal microscopy confirms mitochondria damage and lipid droplets overexpression at late stage TIS

To assess mitochondria damage following DFO treatment, we stained control and DFO-treated Hepg2 cell with MitoTracker and observed the cells under a confocal microscope. The confocal analysis confirmed that treatment with DFO affected mitochondria morphology, with the mitochondrial network visibly losing its organization and mitochondria becoming thin and filamentous (Fig. S2). However, these alterations were only visible after prolonged DFO treatment (7 days), when the senescent phenotype was already fully developed. Differences between control and treated samples were barely visible after 72 hours of treatment. Moreover, confocal microscopy has the added drawbacks of being destructive, necessitating labels that can alter the system and lead to false positives, and not providing a quantitative measure of mitochondria damage.

We assessed DFO-induced lipid droplets (LDs) accumulation by staining of control and DFO-treated Hepg2 cells with BODIPY 493/503 and observing the cells under a confocal microscope. We were able to observe a significant increase in LDs amount and size only after 72h of DFO treatment; the accumulation of LDs was dramatic after 7 days of treatment (Fig. S3). In addition to LDs overexpression not being visible at early TIS stages, confocal microscopy necessitates the labeling of the samples, which can perturb the system and therefore is not suitable for strong quantitative analysis.

Supplementary Note 3: Spontaneous Raman study of CH-stretching vibrational modes of typical cellular lipids

Spontaneous Raman measurements were performed using a home-built confocal Raman microscope. The excitation light provided by a continuous-wave diode laser centered at 660nm (Cobolt AB, Flamenco, Solna, Sweden) was expanded by a beam expander (GBE05-B, ThorLabs, Newton, NJ, USA) and reflected by a single-edge dichroic beamsplitter (Di03-R660-t1-25x36, Semrock, Inc., Rochester, NY, USA) aiming to separate excitation and emitted Raman photons. The light entered the back port of a commercial inverted microscope (IX73, Olympus Europa SE & Co. KG, Hamburg, Germany) and was focused on the sample using a dry/air 50x

objective (MPLFLN50X 20x/0.80 NA, Olympus). The scattered light was collected by the same objective and transmitted by the dichroic beamsplitter. A long-pass (664nm) edge filter (LP02-664RU-25, Semrock, Inc.) was then used to remove the residual laser light and the scattered light was focused by a lens (f=35 mm, AC254-035-B-ML, ThorLabs, Newton, NJ, USA) on the entrance slit of a spectrometer (Isoplane160, Princeton instruments, Trenton, NJ, USA) equipped with a grating of 1200 gr/mm and connected to a front illuminated CCD (PIXIS256F, Princeton Instruments, Trenton, NJ, USA). Wavenumber calibration has been performed using toluene and a ArHg lamp (AvaLight CAL-MINI, Avantes, Apeldorn, The Netherlands) as references. Intensity calibration has been performed using a calibrated white lamp (AvaLight HAL-CAL-MINI, Avantes, Apeldorn, The Netherlands). Lipids liquid at room temperature were studied without further preparation onto a quartz slide; lipids solid at room temperature were diluted in methanol before the Raman analysis, and studied after deposition and drying (24h) of a 1 μL drop onto mirrored stainless-steel slides (Renishaw plc, Wotton-under-Edge, UK). The excitation power for all experiments was kept at 60mW. The spectra were acquired by applying integration time of three seconds and by averaging five acquisitions.

<u>Supplementary Note 4: Generalizability of NLO traits of early TIS – Doxorubicine-induced senescence on HepG2 and TPC1 cell lines</u>

To check the generalizability of results, we conducted multimodal NLO microscopy measurements on different human cancer cell lines, treated with Doxorubicine (Doxo), a gold standard chemotherapy agent. We compared Doxo-doped HepG2 cells (*i.e.*, human hepatic cancer cells, as in the TIS model employed during our main study) and 7-days Doxo-doped TPC-1 cells (i.e., human papillary thyroid carcinoma) to their relative untreated controls. Qualitatively, NLO channels show a clearly similar rearrangement when considering controls and TIS cells. The amount of cell cytosol showing lipid droplets-related SRS signal (2850 cm^{-1} Raman shift) increases in both HepG2 (Fig. S8B) and TPC-1 (Fig. S8D) when Doxo-TIS is present, with respect to controls (Fig. S8A,C). Similarly, mitochondria related TPEF signals aggregate evidently as dense and bright spots inside both the cell lines, whereas they look more broadly and evenly distributed in untreated counterparts.

The overproduction of lipids and contraction of mitochondrial networks in TIS conditions, employing a different anticancer therapy in different human cancer cell lines, supports the robustness of NLO features of TIS, for an early, label-free, and clinically translatable detection of the phenotype at early commitment stages.

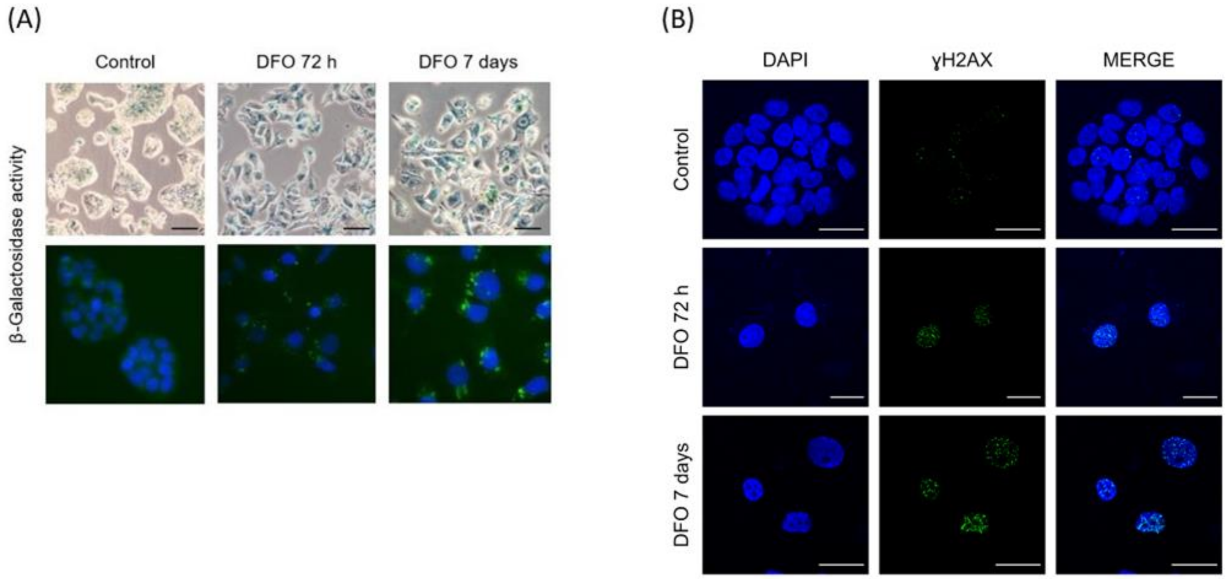

Fig. S1.

Standard β-Galactosidase and yH2AX staining in DFO-doped HepG2 cells. (A) First row: representative images of control, 72 h and 7 days DFO-treated HepG2 cells stained for β-Galactosidase activity (blue). Scale bar: 100 µm. Second row: representative images of control, 72 h and 7 days DFO-treated HepG2 cells stained with BioTracker 519 B-Gal Green Dye, Invitrogen (green) and DAPI (blue). Objective: 40X. **(B)** Representative images of control, 72 h DFO-treated and 7 days DFO-treated HepG2 cells (from top to bottom) stained for nuclei (blue), yH2AX (green), and merged image of both (from left to right). Scale bar: 25 µm.

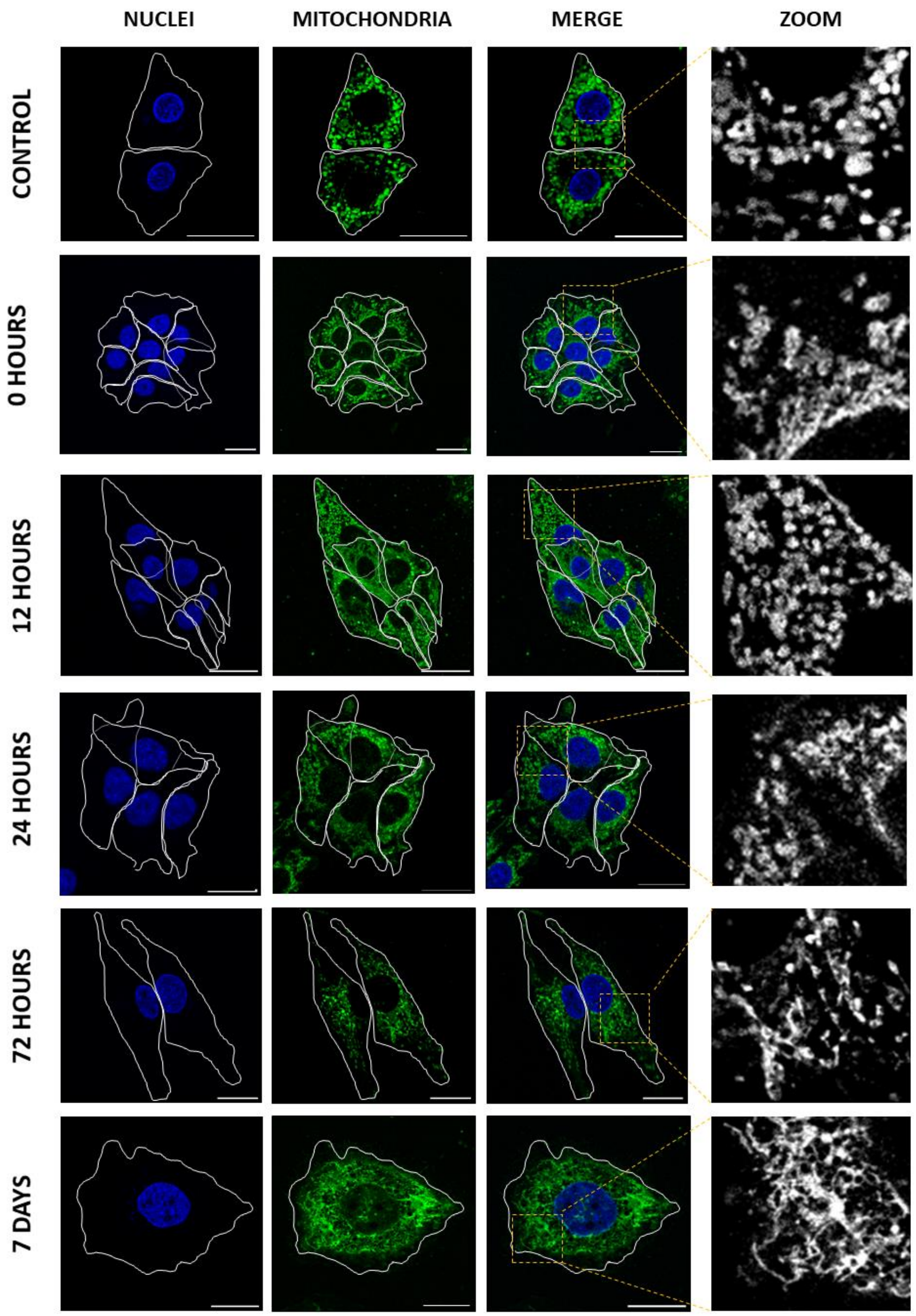

Fig. S2.

Representative images of labeled control and DFO-treated HepG2 cells. Samples were stained for mitochondria (MitoTracker; shown in green) and nuclei (DAPI; shown in blue). The mitochondria network for each condition is shown in detail in the zoomed-in gray-scale images.

The borders of the cells are delineated with a white line. Cells were observed and imaged using a confocal microscope. Scale bar: 25 µm. The control is equivalent to 0 hours of DFO treatment.

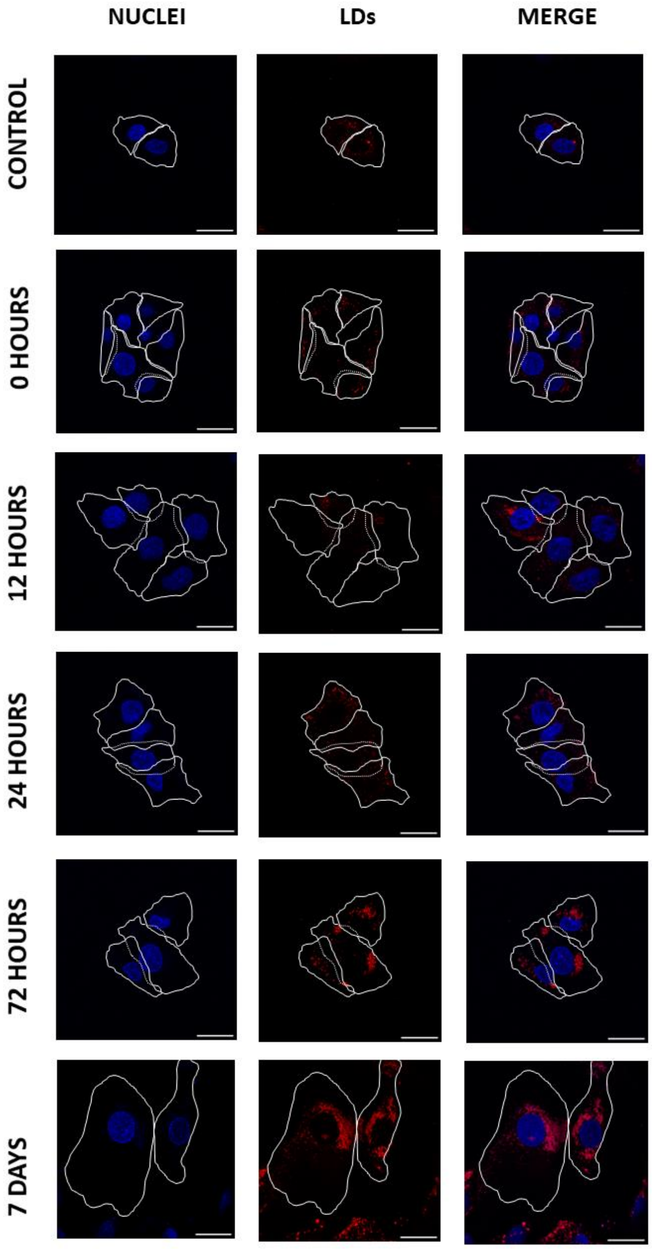

Fig. S3.

Representative images of control and DFO-treated HepG2 cells at different time points. Cells were stained for lipid droplets (BODIPY; shown in red) and nuclei (DAPI; shown in blue). Cells were observed and imaged using a confocal microscope. Scale bar: 25 µm. The control is equivalent to 0 hours of DFO treatment.

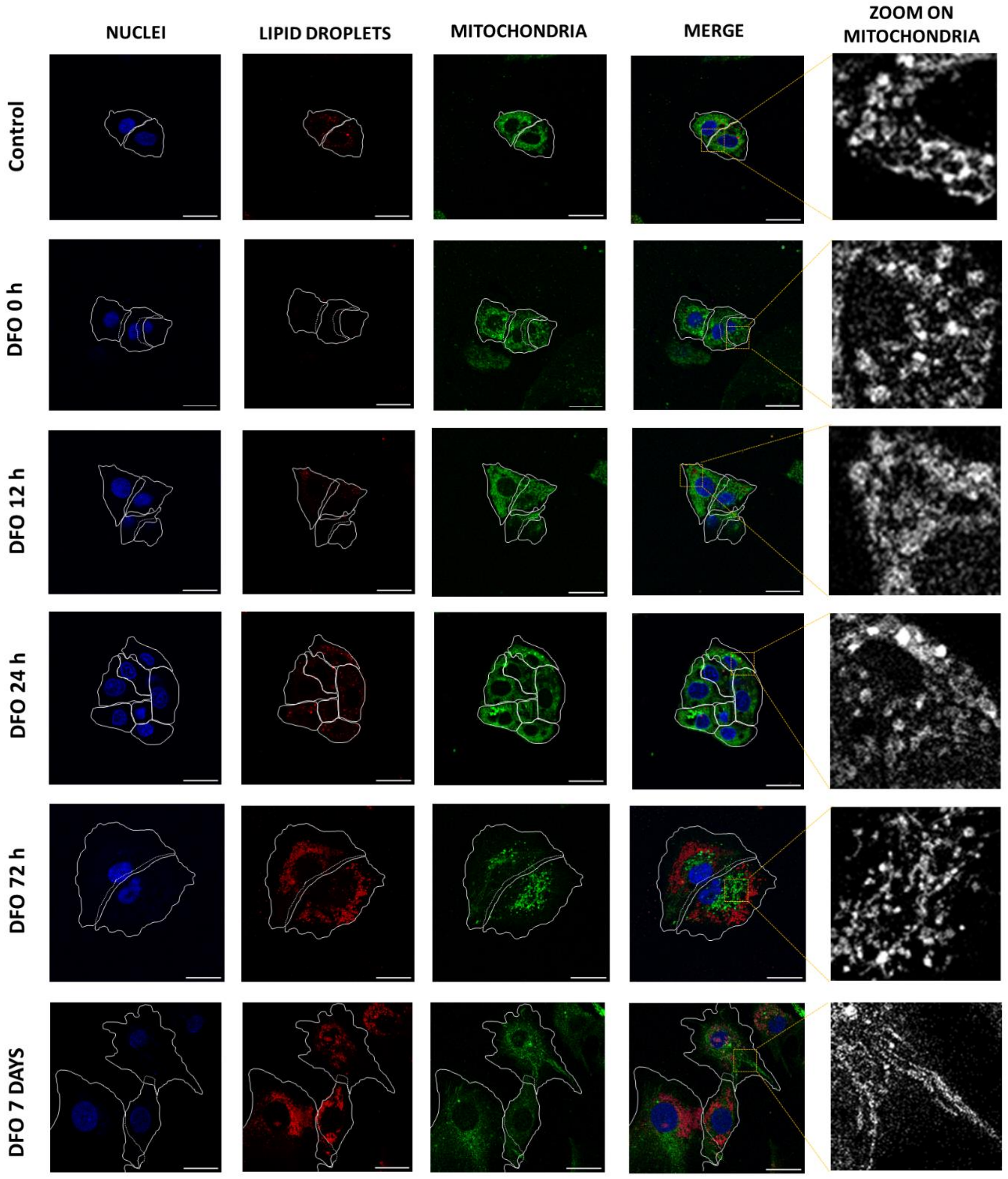

Fig. S4.

Representative images of labeled control and DFO-treated HepG2 cells. Samples were stained for mitochondria (MitoTracker; shown in green), lipid droplets (BODIPY, shown in red), and nuclei (DAPI, shown in blue). The mitochondria network for each condition is shown in detail in the zoomed-in gray-scale images. The borders of the cells are delineated with a white

line. Cells were observed and imaged using a confocal microscope. Scale bar: 25 µm. The control is equivalent to 0 hours of DFO treatment.

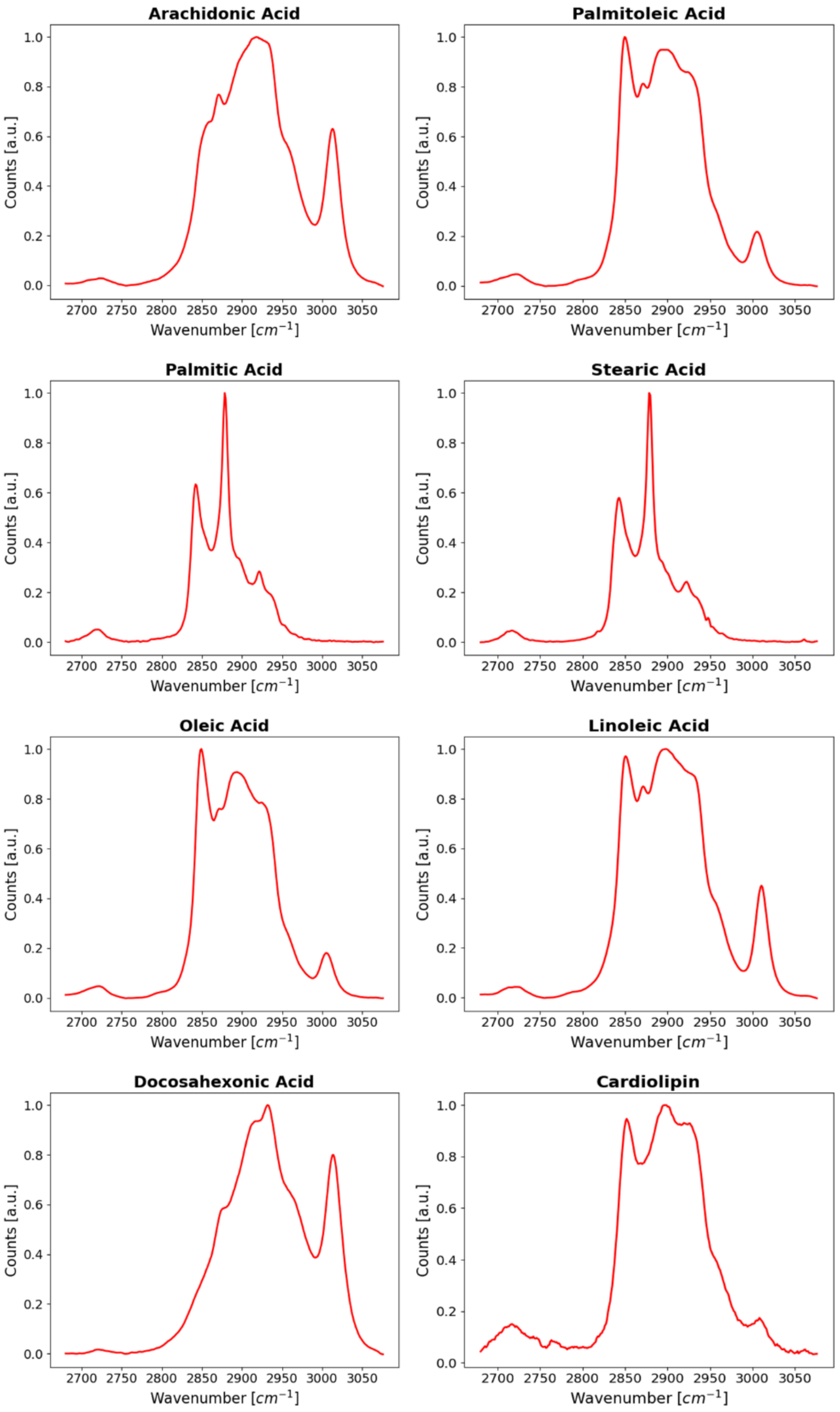

Fig. S5.

Raman spectra in the CH-stretching region of subcellular lipid extracts were employed as standards. The predominant Raman band, 2850 cm^{-1}, corresponds to the symmetric CH_2 stretching in lipid chains, in excellent agreement with the literature (38). This Raman mode was used for coherent Raman investigations in hepatic human cancer cells (HepG2 cell line).

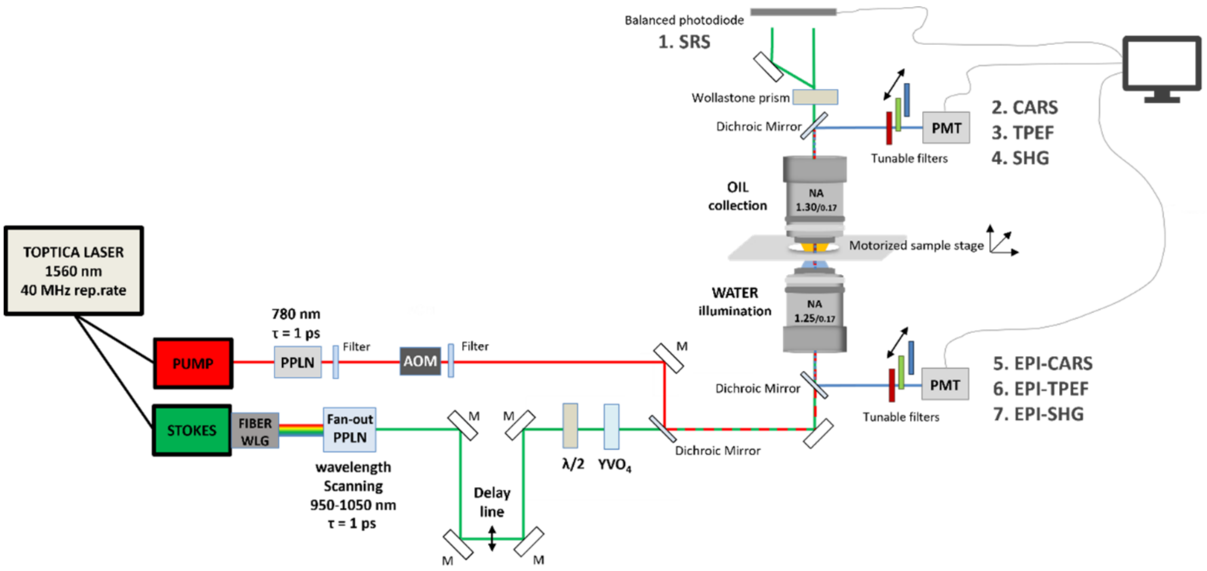

Fig. S6.

Schematics of the multimodal nonlinear optical setup used in the study. It includes Coherent Anti-Stokes Raman Scattering (CARS), Stimulated Raman Scattering (SRS, in the form of SRG at Stokes photon energies), and Two-Photon Excited Fluorescence (TPEF) imaging of HepG2 cells. Additionally, it is designed to detect the Second-Harmonic Generation signals (SHG). The photomultiplier tubes (PMTs) for epi- and forward-detected CARS, TPEF and SHG are preceded by interchangeable optical filters. A 650/40 nm bandpass filter is used for CARS detection (ThorLabs, USA, model: FB650-40). A short pass filter is used to detect TPEF in the range 400 nm - 600 nm (FES0600). For SHG, a 390/18 nm bandpass filter is used (Semrock, USA, model: FF01-390/18-25). The setup includes a module for in-line balanced detection of the SRG signal to minimize the noise in SRS measurements[1]. It requires a time-delayed polarization-multiplexed collinear replica of the Stokes pulse, obtained through a half-wave plate (λ/2) and a birefringent crystal (YVO_4), impinging on the sample at the same position as the Stokes pulse. Such a scheme allows for maintaining a balanced intensity between the probe and reference during image acquisition. The two pulses are separated through a Wollaston prism and detected via a balanced photodiode (BPD). The BPD computes the difference signal and inputs it into a lock-in amplifier (LIA) to demodulate SRG signals, reported in our manuscript as ratios of VRMS signals extracted via LIA and the relative Stokes transmission measured by the BPD (*i.e.*, ΔI/I). WLG: white-light generation via a highly nonlinear fiber to produce tunable Stokes pulses. PPLN: periodically poled lithium niobate crystal for frequency doubling. AOM: acousto-optic modulator operating at 1 MHz. M: mirror. NA: numerical aperture.

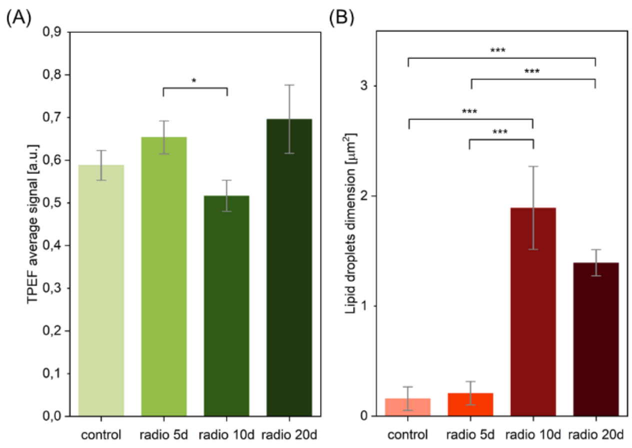

Fig. S7.
Additional indexes from multimodal NLO image analysis of radio-TIS HepG2 cells, over a follow-up period of 20 days after exposure to ionizing radiations. Statistics are reported for the consistency with data in the main manuscript, involving the DFO-doped model of TIS used through the study. **(A)** The bar plot shows a consistent average TPEF signal over time, which is comparable with the slightly increasing signal observed only at later stages in the pro-senescence model (+DFO). **(B)** The bar plot shows an accumulation of lipid vesicles in radio-TIS phenotypes with significantly increasing area, starting at later time points with respect to NAD(P)H and FAD rearrangement. These quantifications confirm our observations of DFO-TIS culture models and support the generalizability of results.

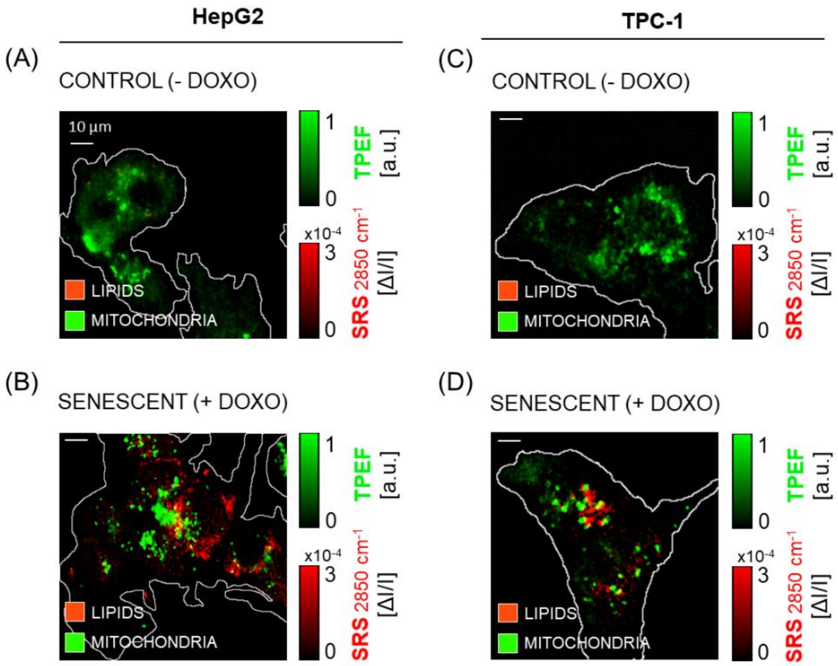

Fig. S8.
Human cancer cells from the HepG2 and TPC-1 cell lines, treated with Doxo as an anticancer therapy for 7 days. Under TIS conditions **(B, D)**, it is evident that significant portions of the cell cytosols feature SRS signal at the CH_2-stretching Raman mode of lipids (shown in red), indicating an overproduction of lipid vesicles is associated with the senescent phenotype. Also, the endogenous TPEF signal is rearranged into bright spots for TIS (shown in green), unlike the widespread and relatively even distribution found in untreated controls **(A, C)**. These results agree with the ones derived from the DFO-doped TIS models employed throughout the main study.

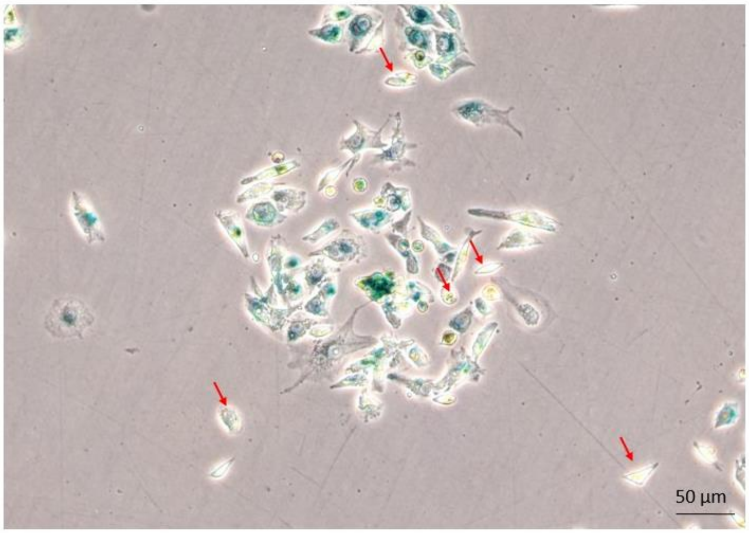

Fig. S9.

A representative image of HepG2 cells after 72 hours of DFO treatment. Cells were stained for β-galactosidase activity (blue). Red arrows indicate dead cells (they are about to detach from the substrate, losing focus, while senescent cells stay fully attached and focused). In our DFO-driven TIS cultures, both healthy (control) and TIS cells adhere to glass or quartz substrates throughout the study (up to 7 days). Conversely, a minority of cells that undergo apoptosis easily detach from the substrate. For both NLO and QPI microscopy measurements, we removed the apoptotic cells via repeated PBS washes prior to imaging. On top of that, TIS cells display evidently different morphology from apoptotic ones: senescent cells appear large, flat and evanescent. In the rare case in which apoptotic cells are not completely wiped off the substrate, they could easily be distinguished as they appeared round and bright (red arrows). Excluding apoptotic cells in our TIS samples from measurements and analyses is a critical and fundamental step to support all the arguments in the presented work.

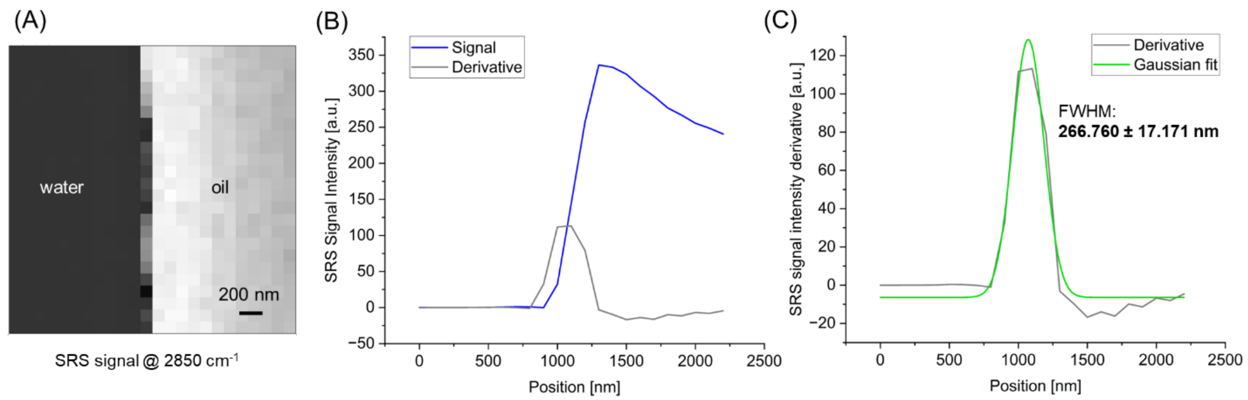

Fig. S10.
Experimental evidence of the maximum lateral resolution of our home built multimodal NLO setup. (A) We evaluated the lateral resolution by exploiting an SRS image of a sharp edge interface between oil (Raman mode at 2850 cm^{-1}) and water, acquired using a pixel dimension of 100 nm. Among the available NLO modalities, SRS is the channel featuring the lower signal-to-noise ratio due to the modulation-transfer detection scheme. Hence, we chose to use an SRS edge image to avoid any experimental resolution overestimation. **(B)** Signal intensity is integrated over columns (in blue) in the image in Figure 2A, and its derivative (grey line). **(C)** Gaussian fit of the signal derivative, featuring a full width at half maximum (FWHM) of 266.760 ± 17.171 nm. We experimentally proved lateral resolution power in line with the theoretical limit obtained through the Rayleigh criterion (~ 280 nm).

	2930 – 2850 cm^{-1} SRS and 2970 cm^{-1} F-CARS
**PCC**	0.110 ± 0.028
**M1**	0.679 ± 0.068
**M2**	0.786 ± 0.057

Table S1. Quantitative colocalization metrics for multimodal NLO image channels of nuclei. Pearson Correlation Coefficient (PCC), and Manders' co-localization coefficients (M1 and M2) for the quantification of co-occurrence of 2930 – 2850 cm^{-1} SRS (protein-rich nuclear areas) versus 2970 cm^{-1} F-CARS (deoxyribose signal). The average PCC value is low, whereas M1 and M2 display modest values: while there is no linear proportionality between the raw 2970 cm^{-1} F-CARS signal of deoxyribose and the SRS subtraction signal, which can be explained considering that there is no raw intensity scaling with the target concentration in the latter, M1 and M2 prove a decent spatial co-occurrence, which is in agreement with visual inspections of nuclei-related NLO images. This analysis further assesses nuclei position in observed cells. Values are displayed as mean ± standard deviation.

Additional Data Explanation (Zenodo repository)

We shared all the quantitative data used through this work in a dedicated open access online repository on Zenodo, https://doi.org/10.5281/zenodo.7848251.

Specifically, we included the data accounting for the following:

- Figure 2. Raw pixel-wise signals detected in NLO images of TIS cells control cells, used to perform colocalization scatterplots and analyses reported. We describe the average colocalization of SRS and F-CARS signals, and TPEF and E-CARS signals, in both phenotypes.
- Figure 4. Raw data from image analyses of TPEF and SRS channels of multimodal NLO images, divided in 5 different time points over the therapy follow-up period. The data describe the early rearrangement of mitochondria (TPEF) and lipid vesicles (SRS) in TIS cells, with respect to control counterparts.
- Figure 6. Raw data from image analyses of QPI images, divided in 4 different time points over the therapy follow-up period. The data describe the early morphological modifications of TIS cells, with respect to control counterparts.
